# Designing Plastrons for Underwater Bubble Capture: From Model Microstructures to Stochastic Nanostructures

**DOI:** 10.1002/advs.202403366

**Published:** 2024-07-02

**Authors:** William S. Y. Wong, Abhinav Naga, Tobias Armstrong, Bhuvaneshwari Karunakaran, Dimos Poulikakos, Robin H. A. Ras

**Affiliations:** ^1^ Department of Applied Physics School of Science Aalto University Espoo FI‐02150 Finland; ^2^ Department of Physics Durham University Durham DH1 3LE United Kingdom; ^3^ Institute for Multiscale Thermofluids, School of Engineering University of Edinburgh Edinburgh EH9 3FD United Kingdom; ^4^ Laboratory for Multiphase Thermofluidics and Surface Nanoengineering Department of Mechanical and Process Engineering ETH Zurich Zurich 8092 Switzerland; ^5^ Laboratory of Thermodynamics in Emerging Technologies Department of Mechanical and Process Engineering ETH Zurich Zurich 8092 Switzerland

**Keywords:** bubble absorption, bubble coalescence, bubble rupture, superhydrophobic, super liquid repellent

## Abstract

Bubbles and foams are often removed via chemical defoamers and/or mechanical agitation. Designing surfaces that promote chemical‐free and energy‐passive bubble capture is desirable for numerous industrial processes, including mineral flotation, wastewater treatment, and electrolysis. When immersed, super‐liquid‐repellent surfaces form plastrons, which are textured solid topographies with interconnected gas domains. Plastrons exhibit the remarkable ability of capturing bubbles through coalescence. However, the two‐step mechanics of plastron‐induced bubble coalescence, namely, rupture (initiation and location) and subsequent absorption (propagation and drainage) are not well understood. Here, the influence of 1) topographical feature size and 2) gas fraction on bubble capture dynamics is investigated. Smaller feature sizes accelerate rupture while larger gas fractions markedly improve absorption. Rupture is initiated solely on solid domains and is more probable near the edges of solid features. Yet, rupture time becomes longer as solid fraction increases. This counterintuitive behavior represents unexpected complexities. Upon rupture, the bubble's moving liquid‐solid contact line influences its absorption rate and equilibrium state. These findings show the importance of rationally minimizing surface feature sizes and contact line interactions for rapid bubble rupture and absorption. This work provides key design principles for plastron‐induced bubble coalescence, inspiring future development of industrially‐relevant surfaces for underwater bubble capture.

## Graphical abstract

To achieve efficient plastron‐induced bubble coalescence, the phenomenon must be understood as the tandem process of 1) bubble rupture, and 2) bubble absorption. Here, the influence of interacting feature size and gas fraction is assessed. The importance of small feature sizes and high gas fraction is essential, alongside an uneven hierarchical texture for rapid bubble rupture and absorption.

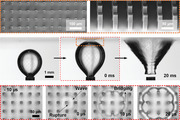

## 1 Introduction

Bubble coalescence is a phenomenon that is of interest to both fundamental and applied investigations.^[^ ^1^, ^2^, ^3^, ^4^ ^]^ Understanding and tuning interactions^[^ ^5^, ^6^, ^7^, ^8^, ^9^, ^10^, ^11^, ^12^ ^]^ between bubbles in bulk foam^[^ ^13^, ^14^, ^15^, ^16^ ^]^ has been essential in many industrial processes. However, chemical additives^[^ ^5^, ^6^ ^]^ and/or mechanical agitation are almost always required. Engineering surfaces^[^ ^17^, ^18^ ^]^ capable of inducing chemical‐free and energetically‐passive bubble coalescence have immense disruptive potential. One promising method involves the use of underwater plastrons (textured solid topographies with interconnected gas domains) for plastron‐induced bubble coalescence.^[^ ^18^, ^19^ ^]^ Unfortunately, this phenomenon still lacks a comprehensive description.

Several fundamental distinctions between the interfacial bubble‐to‐plastron and bulk bubble‐to‐bubble configurations exist. First, a plastron is composed of both solid and gas domains (composite) while bubbles only possess a gas component.^[^ ^20^ ^]^ It remains unclear how rupture initiates on a plastron. Does it rupture on the solid^[^ ^18^, ^19^ ^]^ or gas^[^ ^21^ ^]^ domains? Or both? Second, a plastron has a stiffer interface as the liquid‐solid interface is non‐compressible and the liquid‐gas interface has much smaller curvatures that resist flexing of the (nano‐to‐micro)‐metric menisci. In contrast, macroscopic bubbles have milli‐metric scale curvatures that are comparatively flexible. Third, bubble‐to‐bubble coalescence concludes after the film ruptures as bubbles spontaneously and completely merge. In bubble‐to‐plastron coalescence, absorption of ruptured bubbles occurs via moving contact lines. This entails local liquid‐solid interactions that influence absorption dynamics.

Macroscopically, bubble‐to‐plastron coalescence involves two distinct steps: 1) liquid film rupture,^[^ ^1^ ^]^ followed by 2) bubble absorption.^[^ ^22^ ^]^ The former is a stochastic nanometric (≤ 100 nm) phenomenon^[^ ^16^ ^]^ controlled by interaction forces and contact areas. The latter is a deterministic micrometric (≈10–1000 µm) phenomenon^[^ ^22^ ^]^ defined by wettability and contact lines. A synchronized investigation of both is needed to understand the uniquely coupled phenomena. The thinning of a nanometric liquid film (*h_f_*) leading to bubble rupture is often described by the Stokes–Reynolds equation (e.g., tangentially immobile interface),

(1) $$\frac{\partial h_{f}}{\partial t}=\frac{1}{12\eta r}\frac{\partial }{\partial r}\left(r{h_{f}}^{3}\frac{\partial P}{\partial r}\right)$$

where *r* represents the local lateral dimension, that is, the interaction domain, over which time‐dependent (*t*) film (height *h_f_*) drainage occurs,^[^ ^1^ ^]^ and η represents the liquid viscosity. The pressure difference ($\frac{\partial P}{\partial r}$) includes local deformations described by the augmented Laplace pressure and disjoining pressure, Π(*h_f_*).^[^ ^23^ ^]^ The assumption of tangential mobility or immobility (induced by trace contaminants) influences the pre‐factor of the Stokes–Reynolds (Equation 1)^[^ ^1^ ^]^ but will not influence broad parametric testing described in this work. Here, we investigate the nature of plastron‐induced bubble rupture and absorption via 1) the interacting topographical feature size (*w*  =  2*r*) and 2) air gaps (gas fraction, α). Both factors alter effective disjoining pressure Π(*h_f_*), hydrodynamic drainage ($\frac{\partial h_{f}}{\partial t}$), and post‐rupture absorption. Micropillar topographies (model microstructured surfaces) and nanoparticulate surfaces (hierarchical nanostructured surfaces) complementarily provide a model‐to‐practical understanding.

We discover that plastron‐induced film rupture is encouraged by smaller solid feature sizes (*w*) but occurs solely on solid domains (higher probability at the corners and edges). This is unexpected as the vdW disjoining pressure, Π_*vdW*_(*h_f_*) ∼ *A*_*H*_ (Hamaker^[^ ^24^ ^]^ constant) imposed by the gas domain is an order of magnitude higher than that of the solid domain. Yet, rupture time becomes longer despite increasing solid fraction, highlighting unexpected complexities within thin‐film liquid flow on a 3D geometry. Upon rupture, a surface with a higher gas fraction (α) experiences lower dissipation by the bubble's moving contact line at the liquid‐solid interface, therefore improving viscous‐dominated absorption.

## 2 Results and Discussion

### 2.1 The Nature of Plastron‐Induced Bubble Rupturing

**Figure 1:**
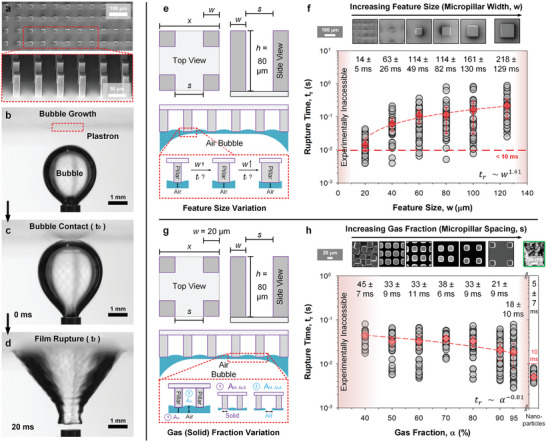
Governing mechanisms in bubble rupture: feature size (*w*) and gas fraction (α). a) Scanning electron micrograph of microstructures. b–d) Plastron‐induced bubble rupture time (*t_r_*) via bubble contact. d) The bubble detaches from the needle, and the plastron begins to absorb it. The two contributions e,g) that drive thin film (bubble) rupture are investigated. The film remains intact in these schematics. First, the e) variable feature size defined by micropillar widths (w) at a fixed gas fraction (α) of 90% assesses the impact of f) interacting structures’ length scales. Second, the g) gas fraction (α) at a fixed feature size (w = 20 µm) assesses the impact of h) gas‐ and/or solid‐ fractions. Thin film stability is governed by *A_H_*, (Hamaker constant) of air‐liquid‐solid (ALS) or air‐liquid‐air (ALA) combinations. *t_r_* is strongly correlated to *w* (f, *t_r_* $∼\;w^{1.41}$) while weakly and inversely correlated to α (h, *t_r_* ∼ α^−0.81^). The non‐experimentally accessible domains (Figures S1 and S2 and Movie S1, Supporting Information) are shaded in light red. Parametric analysis was assessed via statistical rupture (n = 50) in milliQ water, 18.2 MΩ cm. All runs are presented as grey circles while the averages ± standard errors are presented as red diamonds. Temporal resolution of analysis (moving averages) is performed at 0.94 ms.

Micropillar topographies (grid size of ca. 1.2 cm^2^ unless otherwise indicated) are used with a bubble diameter of ca. 4.5 mm (in a contacting oblate profile) in clean milliQ water (18.2 MΩ cm). Micropillars are fabricated by templating polydimethylsiloxane (PDMS, Sylgard 184, w/w 1:10) on a negative SU‐8 mask (maskless lithography, MLA 150, Heidelberg Instruments). Pillars are functionalized with a perfluoroalkylated silane (i.e., 1*H*,1*H*,2*H*,2*H*‐perfluorooctyltrichlorosilane, PFOTS, 97%, Sigma–Aldrich). Pillars heights (*h*) are fixed at 80 µm. Feature sizes (*w*) and gas fractions (α) are studied as independent variables (Figure S1‐2). Collectively, these surfaces are referred to as microstructured surfaces (**Figure** **1**a). A bubble is inflated into contact with the plastron (Figure 1b,c). A pre‐contact velocity of ca. 25–30 mm s^−1^ is registered. As it reaches the plastron, the point of minimum velocity is defined as bubble contact *t*_0_. After a finite period of contact, the thin liquid film separating the bubble ruptures and the bubble is absorbed into the plastron (Figure 1d). This results in a sudden increase in the center‐mass velocity, registering film rupture *t_f_*. The time delay (*t_f_* – *t*_0_) represents the rupture time, *t_r_*. Thin film rupture (Step 1) is stochastic while bubble absorption (Step 2) is deterministic.^[^ ^18^, ^19^ ^]^ For the former, 50 cross‐batch repeats^[^ ^16^, ^18^, ^19^ ^]^ are performed for statistical significance (Figure S3a, Supporting Information). The hydrostatic pressure (1 cm H_2_O/98 Pa), bubble size (4.5 mm), and contact velocity (25–30 mm s^−1^) are kept identical (Figure S4, Supporting Information). Therefore, macroscopic bubble deformations (including dimpling)^[^ ^1^ ^]^ are likely similar in all cases. This isolates our investigation to the parameter spaces belonging to surface properties of the contacting plastron.

Step 1, Rupture Mechanics: Feature Size (*w*) and Gas Fraction (α) Variation

Feature Size (w): To investigate the influence of feature size (*w*) on rupture time (*t_r_*), a series of micropillar topographies is prepared (Figure 1e). The micropillar heights (*h*) are kept at ca. 80 µm, with *w* defined at 20, 40, 60, 80, 100, and 125 µm (Figure 1f **, insets**). The wall‐to‐wall spacing, *s*, between micropillars is configured by fixing gas fraction (α) at 90% at 43, 86, 130, 173, 216, and 270 µm respectively. α is represented either as a fraction (0–1) or a percentage (%). The maximum aspect ratio (*h/w*) is kept at 4 to prevent mechanical instabilities.^[^ ^25^, ^26^ ^]^ Fixing α with variations in *w* assesses the influence of contacting feature sizes on rupture time (Figure 1f).

Despite quantitative limitations^[^ ^1^ ^]^ by analytical models (i.e., Stefan–Reynolds),^[^ ^18^, ^20^ ^]^ an understanding of trends aligning to numerical models (i.e., Stokes–Reynolds)^[^ ^27^ ^]^ can be achieved. For instance, the analytical Stefan–Reynolds model^[^ ^28^, ^29^ ^]^ predicts that *t_r_* $∼\;w^{2}$. Numerical models describing small radii (*w* < 800 µm) film rupture predict a range of *t_r_* −*w^n^* where n is between 1–2.^[^ ^30^ ^]^ In this work, stochastic rupture time analysis (Figure 1f) reveals that, by changing the interacting feature size, *w* from 20–125 µm, *t_r_* varies from 14 ± 5 to 218 ± 129 ms. Average *t_r_* ∼ *w* ^1.41 ^, *R* ^2^ of 0.95. Therefore, predictions provided by current numerical and analytical models can give reasonable trends and estimates for this phenomenon.

The analytical Stefan–Reynolds prediction represents the largest overestimate.^[^ ^18^, ^20^ ^]^ This offset is likely explained by two physical factors. First, Stefan–Reynolds assumes that film thinning occurs evenly over a flat interface (i.e., top of each micropillar). In reality, the liquid film thins unevenly over the top (e.g., dimpling), while also curving^[^ ^31^ ^]^ over pillar edges. The edges are fabricated at a similar length scale regardless of feature size, limited by lithographical resolution (ca. 1 µm). Therefore, the edge effect exists with equal prevalence for both smaller and larger topographies, speeding up rupture. The combination of uneven thinning and an edge effect will consequently diminish the influence of *w*. Second, while α is kept at 90%, differences in pitch distances (*s*) imposed are large: from 43 to 270 µm. A larger pitch results in a larger meniscus (i.e., intruding liquid‐gas interface) between pillars,^[^ ^32^ ^]^ further bolstering the edge effect and reducing the influence of *w*. While a numerical solution is more accurate,^[^ ^30^ ^]^ the standing assumption that film thinning is occurring on a single feature limits its precision (See Supporting Information: Analytical vs Numerical Approximations, Stefan–Reynolds/Stokes–Reynolds). For greater accuracy, a macroscopic non‐axisymmetric model needs to be developed. It should consider a millimetric dimple, micrometric‐to‐nanometric film profiles, asymmetric pillars, and the influence of crossflows (liquid flowing from one pillar to another). This falls beyond the scope of this experimental study but may be of future interest. More details are included in the Supporting Information, under “Other Considerations.”

Gas Fraction (α): A key contributor to plastron‐induced bubble rupture and absorption is the nature of the composited interface. A plastron is composed of both solid (1 − α) and gas (α) fractions, each of which could impose different levels of interactions^[^ ^23^, ^24^, ^33^ ^]^ leading to film drainage and bubble rupture. To date, the corresponding contributions from each component (solid^[^ ^18^ ^]^ or gas)^[^ ^21^ ^]^ on the film thinning behavior are not yet clear. Either has been proposed^[^ ^18^, ^21^ ^]^ for initiating film rupture. To investigate the influence of solid versus gas fraction on bubble rupture (Figure 1g), micropillars are prepared by keeping feature size identical (*w* = 20 µm) at a height of 80 µm, while varying α from 40 to 95% (Figure 1h, **insets**). This variation (Figure 1g) probes the hypothesis of gas and/or solid fraction dependency, as defined by the disjoining pressure equation for vdW interactions (for simplicity, e.g. for a planar interface),^[^ ^33^ ^]^

(2) $$\Pi_{\mathrm{vdW}}=-\frac{A_{H}}{6\pi {h_{f}}^{3}}$$

where *A_H_* is the Hamaker constant and *h_f_* is the film thickness.^[^ ^24^ ^]^ To illustrate the threshold of detection limits, hierarchical nanostructured surfaces are used as controls (Figure 1h, **last column**). The rupture was achieved at an order of magnitude faster (5 ± 7 ms) than microstructured surfaces, nearing the limits of temporal resolution possible here. This does not, however, represent any α dependency (α is likely >> 95%) as nanoparticle clusters also have much smaller^[^ ^18^, ^19^ ^]^ effective feature sizes (*w*). Therefore, while the rupture time recorded from nanostructures may support the trends observed, it remains a speculative conclusion that warrants future investigation.

**Figure 2:**
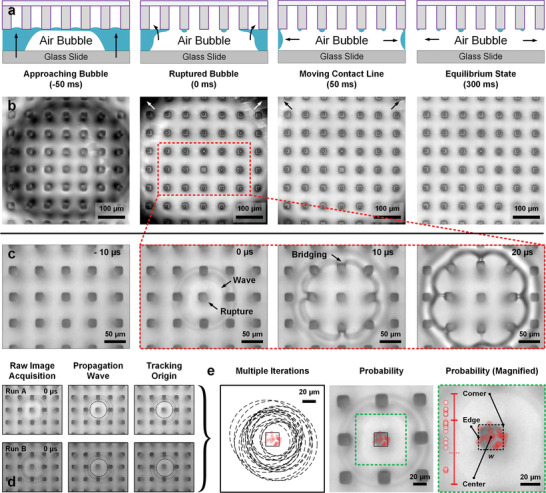
Bubble rupture mechanism: observation through the surface. a,b) A captive bubble on hydrophobized glass approaches (ca. 10 µm s^−1^) micropillars (α of 90%, *w* = 20 µm) until rupture occurs near the apex of the bubble. c) Ultra‐high‐speed imaging (100,000 fps) in the vicinity of rupture captures an outward‐propagating capillary wave, with capillary bridge formation and rupture occurring as it travels. Micropillars do not significantly deform as the wave propagates. d) Tracking the origin of propagation waves (repeats, *n * = 20, Figure S5, Supporting Information) reveals e) ca. 70–75% probability of edge‐corner induced rupture and ca. 25–30% probability of center induced rupture, with 100% occurring on a micropillar. The locations are presented as a statistical line plot of rupture locations versus proximity from the center. The edge and corner are the boundary limits. A histogram is presented in Figure S3b (Supporting Information).

If film rupture is dependent on the solid fraction, increasing α should increase rupture time as there are fewer solid domains (1 − α) on which films rupture. The reverse tests the opposite (gas fraction dependency). Results suggest that a faster rupture time is very weakly correlated (standard errors overlap) to a higher gas fraction (Figure 1h) at *t_r_* ∼ α^−0.81^. However, we acknowledge that the degree of scattering within the data does not support a solid conclusion. Speculatively, this also implies that the rupture event is initiated from the gas domains. However, as we perform a closer examination (see **Figure** **2** and below discussion), the reality runs in contrary. Experimental parametric variations, unlike simulations, incur multi‐parametric consequences. For instance, a higher α also increases *s* which increases the inter‐pillar meniscus curvature and therefore, film thickness (*h_f_*) of menisci between pillars. Per Equation 2, this can severely impact the effective Π_vdW_.

Step 1, Rupture Mechanics: Where and Why?

To understand why rupture time is only weakly correlated to α, a direct microscale visualization of rupture mechanics is performed (Figure 2a,b). By imaging through the surface, real‐time trans‐illuminated brightfield microscopy is used to visualize immersed micropillars (α = 90% and *w *= 20 µm) with an approaching‐rupturing captive bubble. When the thin film separating the bubble from the surface ruptures, an air capillary bridge immediately forms (Figure 2a, 2nd column). This eventually leads to the coalescence of the bubble with the plastron's air volume (Figure 2a, 3rd–4th column). The rupture event repeatedly resulted in a single dry pillar, while a post‐rupture moving contact line deposits an array of microdroplets (Figure 2a,b) over the tops of neighboring micropillars (repeats, *n* = 10). Complete evaporation of microdroplets occurs within ca. 30 s.

To gain an insight into the initial time domain immediately after bubble rupture (*t* < 20 µs), ultra‐high‐speed optical microscopy is performed at 100,000 fps (10 µs temporal resolution, Figure 2c). This enables the visualization and back‐tracing of the propagation wave at a micrometric length scale. Using a circle‐approximation to probabilistically trace the origin of propagation, we observed two different origins for film rupture: 1) Near the edge‐corner of the micropillar (ca. 70–75%) and 2) On the center of the micropillars (ca. 25–30%). This statistical distribution (Figure 2d,e) supports our original hypothesis of the so‐termed edge effect (discussed above).

Most importantly, rupture occurs 100% of the time on the solid domain. This was initially surprising when considering how our experimental findings fit within the theoretical framework. In theory the strongest interaction that drives film rupture is the disjoining pressure, $\Pi_{\mathrm{vdW}}=-\frac{A_{H}}{6\pi {h_{f}}^{3}}$, which is dominated by the Hamaker^[^ ^24^ ^]^ constant, a characteristic property of the sandwiching phases (liquid/gas/solid). Here, *A_H_* influences the disjoining pressure (Figure 1g) via the respective gas and solid fractions. The plastron is composed of fragmented solid (*w*) and gas (*s*) domains. A high destabilizing *A_H_* is desired for film rupture. The perfluoroalkyl‐water‐air, $A_{H}^{FWA}$ (solid) and air‐water‐air, $A_{H}^{AWA}$ (gas) configurations have destabilizing Hamaker^[^ ^24^ ^]^ constants of 1.51 × 10^−21^ and 3.7×10^−20^ J respectively.^[^ ^23^, ^34^, ^35^ ^]^ The destabilizing potential imposed is more than an order higher with the gas domain. However, our direct observations prove that solid interactions dominate even with one‐tenth of the active contact area (10%).

The primary causation behind this unexpected outcome is attributed to the partial intrusion of the liquid‐air interface between microstructures. Under immersion, hydrostatic pressure pushes the flexible air‐water interface inwards. For microstructures, this causes the liquid‐gas film to be thicker (likely ≥ 1 µm)^[^ ^36^ ^]^ than the liquid‐solid film during bubble contact (Figure 2a). Per Equation 2, the disjoining pressure, $\Pi_{\mathrm{vdW}}=-\frac{A_{H}}{6\pi {h_{f}}^{3}}$, scales inversely to the third order with film thickness, *h_f_* but only to the first order with the Hamaker constant, *A_H_*. Therefore, the magnitude of disjoining pressure by gas domains significantly diminishes. It is important to understand how the shape profile of a liquid film on a plastron is not uniform and can be thicker in the regions contacting the gas domains. This, however, also intimately depends on the exact surface profile (*s* and *w*). With microstructures, the physical nature of the film leads to an initially counterintuitive but entirely reasonable observation.

Step 2, Absorption Mechanics: Feature Size (*w*) and Gas Fraction (α) Variation

**Figure 3:**
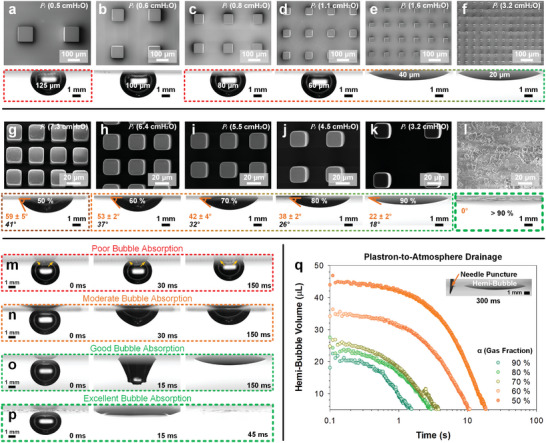
Governing mechanisms to post‐rupture bubble absorption: feature size (*w*) and gas fraction (α). Mechanism 1: Feature size (*w*): a) 125 µm, b) 100 µm, c) 80 µm, d) 60 µm, e) 40 µm, and f) 20 µm were assessed. Mechanism 2: Gas fractions (α): g) 50% (*s *= 8 µm), h) 60% (*s *= 12 µm), i) 70% (*s *= 17 µm), j) 80% (*s *= 25 µm), k) 90% (*s *= 43 µm) at *w* = 20 µm alongside a l) nanostructured variant as the boundary limit. Dynamic time‐dependent bubble rupture and absorption are included for m) poor, n) moderate, o) good, and p) excellent modes of post‐rupture bubble absorption. q) A plastron‐to‐atmosphere connection using needle puncture (inset: 26 G, 260 µm inner diameter) allows for the drainage of hemi‐bubbles, with timescales ranging from 1 to 20 s post‐equilibrium (rupture requires only <0.1 s). Surfaces are at ca. 1 cm underwater.

Feature Size (*w*): After bubble rupture, micropillars with larger *w* (60–125 µm) were unable to absorb the ruptured bubble. Ruptured bubbles maintain near‐spherical profiles at equilibrium, as so‐termed standing bubbles (**Figure** **3**a–d, insets). Micropillars with smaller *w* (20–40 µm) absorb and spread bubbles well, resulting in half‐bubble profiles at equilibrium, as so‐termed hemi‐bubbles (Figure 3e,f, insets). This transition was captured between a *w* of 40–60 µm. In the former (standing bubbles), film rupture occurs discretely, with a gradual “sinking” of the contact line. Film rupture is still clearly observed if compared with an unruptured bubble using Wenzel‐wetted micropillars as a control (plasma‐activated superhydrophilicity, See Movie S2, Supporting Information). The immediately arrested contact line post‐rupture for larger *w* (60–125 µm) is attributed to Wenzel wetting and pinning that occurs at the rim of the bubble after film rupture. This transition at *w* = 60 µm can be explained by considering the impalement pressure (*P_I_*).

The pressure needed for liquids to impale into a unit square (*x*, Figure 1e,g) of micropillars (Figure 3a–f) is defined by $P_{I}=\frac{4}{\sqrt{2}}\frac{\gamma }{\left(w+s\right)}$ where γ is the liquid surface tension in N/m.^[^ ^37^ ^]^ The use of this approximation^[^ ^37^ ^]^ requires sufficiently spaced features, at $\frac{w}{2}\ll \left(w+s\right)$, which is satisfied in all cases. The *P_I_* of each variant is included (in cmH_2_O units) in Figure 3a–f (top right corner). From *w* = 40–60 µm, the characteristic *P_I_* decreases from 1.6 to 1.1 cmH_2_O. Considering that the micropillars are submerged at ca. 1 cm of water (H_2_O) during tests, the Cassie‐state at and beyond 60 µm is metastable. During bubble rupture, contact lines enter domains between pillars, breaking a direct connection to the rest of the plastron. This severed plastron connection is confirmed by the inability to drain the inflated standing bubbles (*w* ≥ 60 µm) after bubble rupture. In contrast, hemi‐bubbles (*w* = 20–40 µm) can be completely drained if the plastron is connected to the atmosphere using a needle puncture, See Movie S3 (Supporting Information). The characteristic drainage behaviors (viscous‐dominated, Blake number < 1, Table S2, Supporting Information) of these hemi‐bubbles are discussed in the Supporting Information: Description of Plastrons and the Drainage of Hemi‐Bubbles. The Blake number (generalized Reynolds number) represents the ratio of inertial to viscous forces during the drainage of fluids through porous media.

Notably, these hemi‐bubbles are not sensitive to small variations in hydrostatic pressure (*P_h_*) imposed by deeper immersion after formation (1–4 cm, i.e., *P_h_* = 98–392 Pa, Figure S6, Supporting Information) nor small variations in overall grid size during formation (From 1.2 to 1.6 cm^2^, a ca. 78% increase in plastron air volume, See Movie S4, Supporting Information). Larger variations will change these observations but remain outside the scope of this study.

Gas Fraction (α), Microstructured Surfaces: To further study the impact of gas fraction on post‐rupture absorption behavior, only stable hemi‐bubbles are considered. This is limited to the most stable configurations (α = 90–50%). In contrast to Wenzel‐pinned standing bubbles observed during feature size *(w)* variation, hemi‐bubbles formed at equilibrium from α = 90–50% remain at the Cassie‐state (Figure 3g–k, insets). The *P_I_* of each variant is included (in cmH_2_O units) at the top right corner of Figure 3g–k for reference. All characteristic *P_I_* increased from 3.2 cmH_2_O (α = 90%) to 7.3 cmH_2_O (α = 50%). Here, a gradual trend in post‐rupture hemi‐bubble geometry is observed. At α = 50%, the equilibrium bubble reflects a moderately absorbed state, with a bubble contact angle of ca. 64°. With increasing α, a lower bubble contact angle is observed at equilibrium, (Figure 3g–k, insets). The equilibrium contact angles can be explained via the Cassie equation,

(3) $$\cos\;\theta_{CB}=\left(1-\alpha \right)\cos\theta_{s}+\alpha \cos\theta_{g}$$

where α and (1‐ α) are the respective gas and solid fractions, while θ_*s*_ and θ_*g*_ are the inherent contact angles on solid and gas respectively. As the wettability of air in the bubble with air in the plastron is perfect, *cos* θ_*g*_ = cos (0)  =  1. Equation 3 reduces to the Cassie–Baxter equation,

(4) $$\cos\;\theta_{CB}=\left(1-\alpha \right)\cos\theta_{s}+\alpha$$

The bubble contact angle of the perfluoroalkylated surface with air, θ_*s*_, is ca. 60° (i.e., complementary to the 120° water contact angle),^[^ ^38^ ^]^ with α = 90%. As such, θ_*CB*_ is 18°. The measured bubble contact angle is ca. 22 ± 2° (Figure 3k, inset). All θ_*CB*_ (in italicized black) is included with the real apparent contact angles, θ_*app*_ (in orange) in Figure 3g–k, insets. Notably, all predicted θ_*CB*_ are ca. 5–20° lower than the average θ_*app*_ (deviation increases to a maximum of 18 ± 5° at α = 50%).

Notwithstanding a limited influence by the plastron's air volume (See above discussion and Movie S4, Supporting Information), the mismatch is largely attributed to the equilibrium pinning of the moving contact line, which keeps a higher θ_*app*_ than expected. We observe in Figure 2b,c that bubble rupture‐absorption results in a moving contact line that depins off each solid micropillar via capillary bridge rupture, leaving microdroplets on top of each pillar. The pinning–depinning dynamics become increasingly dominant with smaller α due to a longer overall effective solid contact line. These dynamics are also visualized via smaller hemi‐bubble profile fluctuations (Figure S7, Supporting Information) and contact line velocities (Figure S8, Supporting Information) post‐rupture at smaller α. Therefore, the partial absorption of the bubble into the plastron never fully reaches the Cassie–Baxter equilibrium due to contact line pinning.

Gas Fraction (α), Hierarchical Nanostructured Surfaces: The hierarchical nanostructured surface studied here illustrates the upper performance limit. Absorption occurs completely, where the contact line rapidly accelerates outward and the entire bubble is absorbed with an apparent contact angle, θ_*app*_ of 0° (Figure 3l). This occurs despite it having a much smaller thickness^[^ ^11^, ^31^ ^]^ and plastron volume than the microstructured surfaces (*h* << 100 µm). The low equilibrium θ_*app*_ of << 10° by nanostructured surfaces bucks the trend observed with microstructured surfaces, where θ_*CB*_ appears higher than the expected θ_*app*_. Fumed nanosilica's bulk porosity (ε) is ca. 95.1–99.3%,^[^ ^39^ ^]^ which predicts a range of θ_*CB*_ = 5°–13°. With heterogeneous nanostructures,^[^ ^40^, ^41^, ^42^ ^]^ the effective gas contact fraction (α) for nanostructured interfaces will slightly vary from ε (surface vs bulk).

The dynamic time‐dependent bubble absorption of poorly‐absorbing surfaces (*w* = 125 µm, α = 90%, Figure 3m), moderately‐absorbing (*w* = 20 µm, α = 50%, Figure 3n), good‐absorbing (*w* = 20 µm, α = 90%, Figure 3o), and excellently‐absorbing (*w* ≈100 nm, α > 90%, Figure 3p) surfaces are included for reference. For the microstructured surfaces, all hemi‐bubbles (Figure 3g–k, insets) can be absorbed via a needle puncture (Figure 3q, Movie S5, Supporting Information), but the viscous‐drainage of air to the atmosphere requires timescales of 10^1^–10^3^ longer than that taken to establish the original profiles (1–20 s vs ≤ 50 ms). For details, see Supporting Information: Inertial versus Viscous Drainage – Blake Number. In contrast to this, nanostructured surfaces do not require any atmospheric connection (See Supporting Information: Micro vs Nanostructuring) to completely (i.e., visibly) absorb the bubble. To describe these contrasting observations, differences in contact line dynamics for microstructured versus hierarchical nanostructured surfaces will now be discussed.

### 2.2 Micro to Nano: Understanding Contact Line Advancement and Pinning in Bubble Absorption

**Figure 4:**
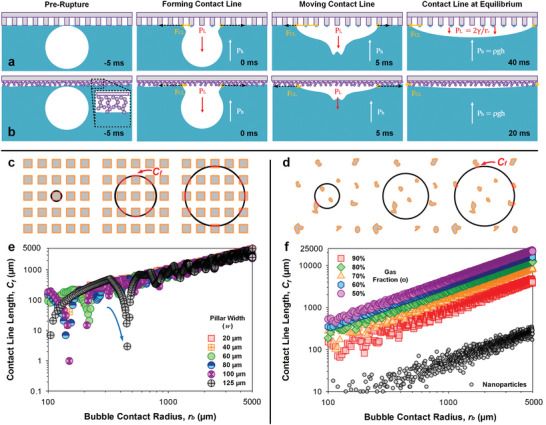
Micro versus nanostructures: simulating contact line motion. Schematized behavior of contact line progression on a) a microstructured surface and b) a nanostructured surface. Schematics are not to scale. The effective solid contact line (red) of the spreading bubble on c) computationally‐populated microstructural or d) experimentally‐extracted nanostructural contacts can be simulated with an axisymmetrically spreading bubble (i.e., radius, *r_b_* = 100–5000 µm). The simulated effective solid contact line (expressed in µm) under e) feature size (*w*) variation and f) gas fraction (α) variation is presented. f) Semi‐empirical mapping of contact domains with the hierarchical nanostructured surface is presented (small grey circles).

To understand the different hemi‐bubble profiles observed, we now explore the time and spatial domains between the point of rupture and the equilibrium state (ca. ≤ 50 ms). During the process of absorption (**Figure** **4**a,b), the dynamic bubble profile is controlled by three contributions. First, hemi‐bubbles are immersed at ca. 1 cm H_2_O, which imposes a static hydrostatic pressure, *P_h_*, of ca. 98.1 Pa. Changes to *P_h_* (Figure S6, Supporting Information) does not influence profiles as it will act on both the hemi‐bubble and the plastron. Second, the overpressure inside the hemi‐bubble (and by extension, the plastron) at equilibrium is defined by its radius of curvature, *r_c_*, as the Laplace pressure (Table S1, Supporting Information). *P_L_* ranges from 8.6 Pa (α = 90%) to 34.6 Pa (α = 50%).

We now also know that a plastron‐to‐atmosphere connection still requires 1–20 s to completely drain hemi‐bubbles. This is 10^1^–10^3^ times longer than the time (ca. 50 ms) needed to reach the equilibrium hemi‐bubble profiles (Figure 3q, Movie S5, Supporting Information). Therefore, a third dominant (yet fast‐acting) contribution must result in these profiles. This contribution comes from forces acting on the contact line (*F_CL_*) as it progresses towards the Cassie–Baxter equilibrium. During contact line motion leading to the equilibrium state (ca. ≤ 50 ms), energy is dissipated by two mechanisms.

First, the contact line progresses at capillary wave velocities (ca. 0.5–2 m s^−1^, Figure S8, Supporting Information) until equilibrium (*t_eq_*). This results in viscous dissipation,

(5) $$E_{v}=\int_{0}^{t_{eq}}C_{f}f\eta U^{2}dt$$

where η is the dynamic viscosity and *U* is the contact line velocity (close to capillary wave speed).^[^ ^43^, ^44^, ^45^, ^46^ ^]^ *f* is a interface‐dependent friction factor which is a constant for similar surfaces.^[^ ^46^ ^]^ *C_f_* is the instantaneous length of the bubble circumference in solid contact, the so‐termed effective contact line length.

Second, as the contact line moves over the micropillars, energy dissipation occurs when capillary bridges rupture while depinning from micropillars. Remnant microdroplets are left on the micropillars (as observed in Figure 2b). The energy dissipated^[^ ^47^ ^]^ during depinning,

(6) $$E_{p}=\pi r_{b}^{2}nW_{p}$$

where $W_{p}=\int_{0}^{\delta_{c}}F_{p}$ *d*δ. *r_b_* is the radius of the hemi‐bubble and *n* is the number of micropillars per unit area. *F_p_* =  4*w*γsin(φ) is the circumference‐defined vertical capillary force component, where φ represents the complementary angle to θ_*rec*_ ≈ 90° for perfluoroalkylated solids.^[^ ^41^, ^42^, ^48^, ^49^ ^]^ The critical rupture extension of the capillary bridge (in the normal direction) is, $\delta_{c}=\frac{w}{2}\;\left\{ln[\frac{8\kappa }{w}]-0.5772\right\}$ (See Supporting Information).^[^ ^50^, ^51^ ^]^

Therefore, the moving contact line experiences 1) viscous dissipation proportionate to the effective contact line length ($E_{v}\;∼\;C_{f})$ and 2) capillary dissipation proportionate to the contact area ($E_{p}\;∼\;nr_{b}^{2}\;∼\;{C_{f}}^{2}$). Further details are included in Supporting Information: Energy Dissipation of the Moving Contact Line. Differences in dissipative losses between variable gas fractions (α) manifest in different contact line velocities (Figure S8, Supporting Information). Eventually, upon nearing equilibrium profiles defined by the Cassie–Baxter state, an offset persists due to pinning at the edge of the contact line close to the final state (≈*C_f_*).

On microstructured surfaces, the effective solid contact line, *C_f_*, is small immediately after bubble rupture and *E* is thus small. Therefore, the contact line velocity is high (Figure 4a, panel 1). As the bubble spreads, *C_f_* increases and *E* increases (Figure 4a, panel 2–3). Contact line velocities thus slow until the equilibrium profile approaches (Figure 4a, panel 4; Figure S8, Supporting Information). On hierarchical nanostructured surfaces, *C_f_* is much smaller due to height differences between each surface agglomerate, thus effectively lowering liquid‐solid interfacial contact (Figure 4b, panel 1–4). To quantitatively illustrate differences between these surfaces, a 2D simulation that predicts *C_f_* is proposed. A script tracks simulated liquid‐solid contact lines of a spreading bubble (assumed axisymmetric) on both microstructured (Figure 4c) and hierarchical nanostructured (Figure 4d) surfaces (See Supporting Information: MATLAB Grid Array and Contact Line Computation). At the high contact line velocities (ca. 0.1–1 m s^−1^) experimentally observed, we assume minimal sagging^[^ ^36^ ^]^ of the interface. The fractional solid contact line analysis (*C_f_*/2π*r_b_*) is also included in Figure S9 (Supporting Information) for reference.

Microstructured Surfaces: First, we study the effect behind variable feature sizes, *w* = 20–125 µm at α = 90% (Figure 4c,d). With larger *w*, the instantaneous *C_f_* occasionally dips (grey, purple, blue, and green circles, Figure 4e). These are regions where the receding contact line, $\frac{dC_{f}}{dr_{b}}$ (Figure 4e, blue arrow), is not supported by significant solid contact, highlighting domains where a collapse into the Wenzel‐state is possible. This supports our experimental observations on impalement with *w*‐dependent transitional wetting (60 µm: green, 80 µm: blue, 100 µm: purple, and 125 µm: grey). Second, we study the effect behind variable gas fractions, α = 50–90% at *w* = 20 µm (Figure 4f). A smaller α leads to a larger *C_f_* but a more stable (lower $\frac{dC_{f}}{dr_{b}}$) contact line progression behavior owing to the denser features (Figure 4f). However, dissipated energy (*E_v_* + *E_p_*) increases with decreasing α, explaining the slower contact line motion that is experimentally observed (Figure S8, Supporting Information).

At equilibrium, we predict how identical α results in a nearly identical *C_f_* regardless of feature size *w* (Figure 4e, at *r_b_* > 3000 µm). This results in similar pinning forces (≈ *C_f_*) that stop the contact line from further motion. This supports our experimental observations behind how equilibrium hemi‐bubble profiles are identical with different *w* (Figure 3e,f, insets). Alternatively, a smaller α leads to a higher *C_f_* and final pinning force (Figure 4f, at *r_b_* > 3000 µm). As a result, an increasing offset from the thermodynamically defined Cassie‐Baxter state occurs with decreasing α, supporting our experimentally observed deviations in bubble contact angles (Figure 3g–k, insets).

Hierarchical Nanostructured Surfaces: In the context of nanostructured surfaces, simulating actual contact domains is not trivial due to uneven liquid‐solid contact. However, prior studies have discussed the phenomenon of contact‐induced pinning and remnants of non‐volatile liquid microdroplets on such surfaces.^[^ ^52^, ^53^, ^54^ ^]^ Notwithstanding minor differences in contact line velocity, such behavior is analogous to our observations with microstructured surfaces (Figure 2). Therefore, confocal imaging of these microdroplet‐decorated surfaces is used to approximate and map contacting domains (Figure S10a,b, Supporting Information). Thereafter, the contact size and density are used to repopulate the simulation grid for estimating *C_f_* during bubble contact line motion (Figure S10c–f, Supporting Information). With the use of spray‐deposited fumed silica nanoparticles, the *C_f_* and *C_f_* fraction (Figure 4f; Figure S9b, Supporting Information, small grey circles) are more than an order smaller than the finest microstructured surfaces (*w* = 20 µm, *s* = 43 µm). During bubble spread, the much smaller *C_f_* experiences lesser dissipation which likely results in a faster contact line progression (Figure S8, Supporting Information). Notably, a peak velocity of ca. 2 m s^−1^ is achieved versus just ca. 0.5 m s^−1^ with microstructured surfaces.

At equilibrium, the *C_f_* fraction is only 0.5–1.1%. If we consider the *C_f_* fraction as the effective solid contact fraction (1‐α), the predicted θ_*CB*_ falls between the range of 4–6°. Notably, this effective gas contact fraction (α) of ca. 98.9–99.5% appears to approach the upper limits of nanosilica's known bulk porosity of ca. 95.1–99.3%.^[^ ^39^ ^]^ To provide an intuitive understanding of other nanostructured surfaces, the *C_f_* profiling of nanofilaments^[^ ^55^ ^]^ and soot‐templated^[^ ^56^ ^]^ nanostructures gives a *C_f_* fraction ranging from ca. 0.5–2.0% (Figure S11, Supporting Information). Note that the actual *C_f_* fraction may still be higher than the results from our simulations as microdroplet remnants tend to be an underestimate of actual contact (Figure 2b).

### 2.3 Self‐Propelled Underwater Bubbles

**Figure 5:**
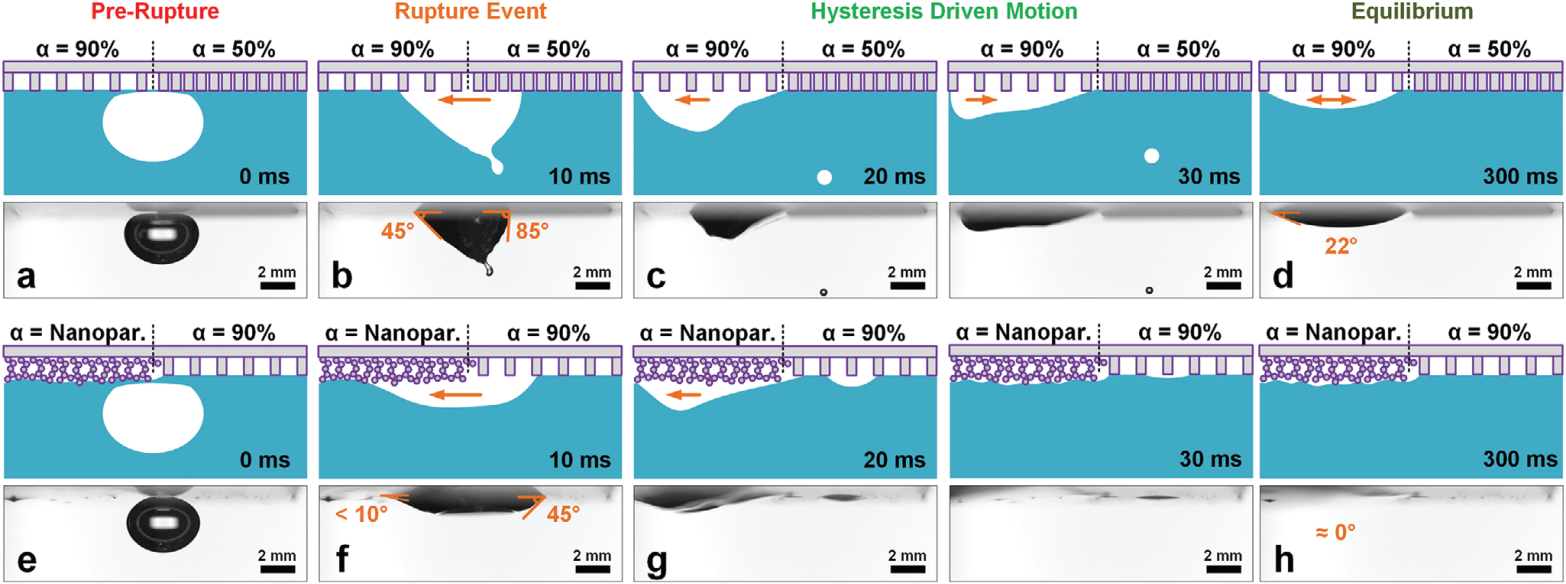
Self‐propelled underwater bubbles on split‐grids. Using sharp boundary transitions (a–d: α = 90% vs 50%) or (e–h: α = 90% vs Nanoparticles), a bubble can be made to a,e) rupture and propel itself across the boundary using b,f) wetting‐hysteresis driven forces at ca. a–d) 0.29 m s^−1^ or e–h) 0.44 m s^−1^ before reaching d) equilibrium. Schematics are not to scale.

With the new understanding behind how rupture‐and‐absorption occurs, we now show how plastron‐induced bubble rupture is phenomenologically unique from currently known wetting behaviors. A micropillar array is fabricated by aligning two grids of α = 90% versus 50% (*w* = 20 µm) (**Figure** **5**a, so‐termed split grid). A bubble is then ruptured by contacting the split. A notably higher bubble contact angle forms on α = 50%, at ca. 85° while a lower bubble contact angle forms on α = 90%, at ca. 45° (Figure 5b). Differences in the mobility of contact line and spontaneity in spreading leads to the asymmetric bubble shape. Per Equation 6, capillary‐induced depinning dissipation is 20–30 times higher ($\approx \;C_{f}^{2}$) on α = 50% than α = 90% (Figure 4f, at *r_b_* ≥ 2000 µm). The bubble is propelled completely off the grid with α = 50% (Figure 5c,d; Movie S6, Supporting Information) and settles at equilibrium on the grid with α = 90%. When competition is presented between α = 90% and *“nano‐structured”* surfaces, a similar behavior arises, albeit differences in velocity and contact angles. In this case, however, the bubble is propelled into the latter (Figure 5e–h). When compared to sliding water drops (in air),^[^ ^57^, ^58^ ^]^ the directed motion is visually reversed, moving toward the side with a lower contact angle. In both cases (Figure 5b,f), the differences in the sliding bubble's contact angles (in water) between the higher and lower side are ca. 35–40°.

Notably, when compared to current observations with microstructural gradients, such self‐propelled motion is unexpected (Movies listed in Supporting Information). 1) A water drop on a plasma‐treated superhydrophilic split‐grid (i.e., the in‐air analog) experiences Lucas–Washburn styled wicking^[^ ^59^ ^]^ but the contact line remains pinned (Movie S7, Supporting Information). 2) Replacement of the air bubble with a floating hexane drop shows how its contact line also remains pinned upon rupture of the thin water film (Movie S8, Supporting Information). 3) When replacing the split‐grid with a linear gradient profile (from ca. *s* < 10 µm to *s* = 100 µm, Δ*s* = 0.25 µm, *w* = 20 µm), no sliding bubble motion occurs, likely due to insufficient surface energy contrasts (Movie S9, Supporting Information). To achieve topographically induced self‐propulsion, we require 1) sharp transitions for overcoming the energy threshold of pinning (Figure 5), and 2) a target phase with low density and viscosity (i.e., gases in bubbles vs liquids in drops).

## 3 Conclusions

The rational design of surfaces for bubble capture (rupture‐and‐absorption) is of significant interest to applications involving bubble and froth control. Using model microstructured surface topographies, we show that bubble rupture speeds up with decreasing feature size (*w*). The bubble rupture time, (*t_r_*) scales with *t_r_* ∼ *w* ^1.41^. We then demonstrate and explain how lower liquid‐solid interfacial contact (1 − α) can significantly improve the spontaneous absorption of a ruptured bubble. This is attributed to lower energy dissipated by the moving contact line as the bubble spreads and lower contact line pinning as spreading nears equilibrium. Our findings illustrate why hierarchical nanostructures are still superior in bubble rupture‐and‐absorption. They have 1) very fine feature sizes (*w* < 100 nm) while also 2) possessing very low liquid‐solid interfacial contact (0.5–1.1%). Together, these characteristics confer excellent bubble rupture‐and‐absorption properties. Our study unravels the underlying physics governing plastron‐induced bubble rupture‐and‐absorption, enabling us to provide a comprehensive surface design guide for achieving passive and efficient bubble capture. With future improvements in lithographical and 3D‐printing resolution, “designer micro‐to‐nanostructured surfaces” may eventually replace the use of stochastic nanomaterials.

## 4 Experimental Section

Details of all experiments are included in the Supporting Information (Synthesis and Characterization).

## Conflict of Interest

The authors declare no conflict of interest.

## Author Contributions

W.S.Y.W. designed the experiments, analyzed the data, and prepared the manuscript. B.K. performed some of the lithography optimization processes to confirm cross‐testing reproducibility. A.N. helped to verify the analytical and numerical arguments. T.N. and D.P. aided in the ultra‐high‐speed analysis of the rupture phenomenon, focusing on understanding where, why, and how the rupture event took place. W.S.Y.W planned and wrote the manuscript. R.H.A.R. verified the outline of the manuscript. All authors reviewed and approved the manuscript.

## Supporting information

Supporting Information

Supplemental Movie 1

Supplemental Movie 2

Supplemental Movie 3

Supplemental Movie 4

Supplemental Movie 5

Supplemental Movie 6

Supplemental Movie 7

Supplemental Movie 8

Supplemental Movie 9

Supplemental Movie 10

Supplemental Movie 11

Supplemental Movie 12

Supplemental Movie 13

## Data Availability

The data that support the findings of this study are available in the supplementary material of this article.

## References

[1] D. Y. C. Chan , E. Klaseboer , R. Manica , Soft Matter 2011, 7, 2235. 10.1039/c5sm03151f 26924623

[2] A. Dippenaar , Int. J. Miner. Process. 1982, 9, 15.

[3] A. Dippenaar , Int. J. Miner. Process. 1982, 9, 1.

[4] J. D. Paulsen , R. Carmigniani , A. Kannan , J. C. Burton , S. R. Nagel , Nat. Commun. 2014, 5, 3182. 24458225 10.1038/ncomms4182

[5] D. Langevin , Curr. Opin. Colloid Interface Sci. 2015, 20, 92.

[6] J. Lu , C. M. Corvalan , Y. M. J. Chew , J.‐Y. Huang , Chem. Eng. Sci. 2019, 196, 493.

[7] S. Ben , Y. Ning , Z. Zhao , Q. Li , X. Zhang , L. Jiang , K. Liu , Adv. Funct. Mater. 2022, 32, 2113374.

[8] S. Zhu , Y. Bian , T. Wu , C. Chen , Y. Jiao , Z. Jiang , Z. Huang , E. Li , J. Li , J. Chu , Y. Hu , D. Wu , L. Jiang , Nano Lett. 2020, 20, 5513. 32539420 10.1021/acs.nanolett.0c02091

[9] J. Zhang , P. Liu , B. Yi , Z. Wang , X. Huang , L. Jiang , X. Yao , ACS Nano 2019, 13, 10596. 31465692 10.1021/acsnano.9b04771

[10] A. M. Rather , Y. Xu , Y. Chang , R. L. Dupont , A. Borbora , U. I. Kara , J.‐C. Fang , R. Mamtani , M. Zhang , Y. Yao , S. Adera , X. Bao , U. Manna , X. Wang , Adv. Mater. 2022, 34, 2110085. 10.1002/adma.202110085 35089623

[11] H. Zhan , Z. Yuan , Y. Li , L. Zhang , H. Liang , Y. Zhao , Z. Wang , L. Zhao , S. Feng , Y. Liu , Nat. Commun. 2023, 14, 6158. 37789018 10.1038/s41467-023-41918-y PMC10547833

[12] M. Hu , F. Wang , L. Chen , P. Huo , Y. Li , X. Gu , K. L. Chong , D. Deng , Nat. Commun. 2022, 13, 5749. 36180429 10.1038/s41467-022-33424-4 PMC9525293

[13] D. Tao , Sep. Sci. Technol. 2005, 39, 741.

[14] R. F. Tabor , D. Y. C. Chan , F. Grieser , R. R. Dagastine , Angew. Chem., Int. Ed. 2011, 50, 3454. 10.1002/anie.201006552 21400646

[15] Y. Xing , X. Gui , L. Pan , B.‐E. Pinchasik , Y. Cao , J. Liu , M. Kappl , H.‐J. Butt , Adv. Colloid Interface Sci. 2017, 246, 105. 28619381 10.1016/j.cis.2017.05.019

[16] B. Liu , R. Manica , Q. Liu , E. Klaseboer , Z. Xu , G. Xie , Phys. Rev. Lett. 2019, 122, 194501. 31144923 10.1103/PhysRevLett.122.194501

[17] K. I. Hegner , W. S. Y. Wong , D. Vollmer , Adv. Mater. 2021, 33, 2101855. 10.1002/adma.202101855 PMC11468632 34365676

[18] W. S. Y. Wong , A. Naga , L. Hauer , P. Baumli , H. Bauer , K. I. Hegner , M. D'Acunzi , A. Kaltbeitzel , H.‐J. Butt , D. Vollmer , Nat. Commun. 2021, 12, 5358. 34504098 10.1038/s41467-021-25556-w PMC8429590

[19] K. I. Hegner , W. S. Y. Wong , D. Vollmer , Adv. Mater. 2021, 33, 2101855. 10.1002/adma.202101855 PMC11468632 34365676

[20] A. Cassie , S. Baxter , J. Chem. Soc. Faraday Trans. 1944, 40, 546.

[21] C. Shi , X. Cui , X. Zhang , P. Tchoukov , Q. Liu , N. Encinas , M. Paven , F. Geyer , D. Vollmer , Z. Xu , H.‐J. Butt , H. Zeng , Langmuir 2015, 31, 7317. 26065326 10.1021/acs.langmuir.5b01157

[22] H. de Maleprade , C. Clanet , D. Quéré , Phys. Rev. Lett. 2016, 117, 094501. 27610858 10.1103/PhysRevLett.117.094501

[23] M. Kappl , H. J. Butt , Surface and interfacial forces, Wiley, Hoboken, New Jersey 2018.

[24] H. C. Hamaker , Physica 1937, 4, 1058.

[25] L. Aoun , P. Weiss , A. Laborde , B. Ducommun , V. Lobjois , C. Vieu , Lab Chip 2014, 14, 2344. 24836927 10.1039/c4lc00197d

[26] Y. Zhang , C.‐W. Lo , J. A. Taylor , S. Yang , Langmuir 2006, 22, 8595. 16981781 10.1021/la061372+

[27] G. K. Batchelor , An introduction to fluid dynamics, Cambridge University Press, Cambridge, 2000.

[28] O. Reynolds , Philos. Trans. R. Soc. 1886, 177, 157.

[29] O. Reynolds , Philos. Trans. R. Soc. 1997, 177, 157.

[30] M. S. Shah , C. R. Kleijn , M. T. Kreutzer , V. van Steijn , Phys. Rev. E: Stat. Phys., Plasmas, Fluids, Relat. Interdiscip. Top. 2021, 6, 013603.

[31] F. Schellenberger , N. Encinas , D. Vollmer , H.‐J. Butt , Phys. Rev. Lett. 2016, 116, 096101. 26991185 10.1103/PhysRevLett.116.096101

[32] A. Tuteja , W. Choi , G. H. McKinley , R. E. Cohen , M. F. Rubner , MRS Bull. 2008, 33, 752.

[33] J. N. Israelachvili , in Intermolecular and surface forces (third edition), (Ed: J. N. Israelachvili ), Academic Press, San Diego, 2011, pp. 253–289.

[34] D. Tabor , R. H. S. Winterton , Proc Math Phys Eng Sci 1969, 312, 435.

[35] E. M. Lifshitz , M. Hamermesh , in Perspectives in theoretical physics, (Ed: L. P. Pitaevski ), Pergamon, Oxford, United Kingdom 1992, pp. 329–349.

[36] P. Papadopoulos , L. Mammen , X. Deng , D. Vollmer , H.‐J. Butt , Proc. Natl. Acad. Sci. USA 2013, 110, 3254. 23382197 10.1073/pnas.1218673110 PMC3587223

[37] H.‐J. Butt , C. Semprebon , P. Papadopoulos , D. Vollmer , M. Brinkmann , M. Ciccotti , Soft Matter 2013, 9, 418.

[38] T. Nishino , M. Meguro , K. Nakamae , M. Matsushita , Y. Ueda , Langmuir 1999, 15, 4321.

[39] V. M. Gun'ko , I. F. Mironyuk , V. I. Zarko , E. F. Voronin , V. V. Turov , E. M. Pakhlov , E. V. Goncharuk , Y. M. Nychiporuk , N. N. Vlasova , P. P. Gorbik , O. A. Mishchuk , A. A. Chuiko , T. V. Kulik , B. B. Palyanytsya , S. V. Pakhovchishin , J. Skubiszewska‐Zięba , W. Janusz , A. V. Turov , R. Leboda , J. Colloid Interface Sci. 2005, 289, 427. 16024031 10.1016/j.jcis.2005.05.051

[40] A. Marmur , E. Bittoun , Langmuir 2009, 25, 1277. 19125688 10.1021/la802667b

[41] G. McHale , Langmuir 2007, 23, 8200. 17580921 10.1021/la7011167

[42] L. Gao , T. J. McCarthy , Langmuir 2007, 23, 3762. 17315893 10.1021/la062634a

[43] D. Bonn , J. Eggers , J. Indekeu , J. Meunier , E. Rolley , Rev. Mod. Phys. 2009, 81, 739.

[44] P. G. de Gennes , Rev. Mod. Phys. 1985, 57, 827.

[45] J. H. Snoeijer , B. Andreotti , Annu. Rev. Fluid Mech. 2013, 45, 269.

[46] A. Carlson , M. Do‐Quang , G. Amberg , J Fluid Mech 2011, 682, 213.

[47] H.‐J. Butt , N. Gao , P. Papadopoulos , W. Steffen , M. Kappl , R. Berger , Langmuir 2017, 33, 107. 28001428 10.1021/acs.langmuir.6b03792

[48] W. S. Y. Wong , M. S. Kiseleva , S. Zhou , M. Junaid , L. Pitkänen , R. H. A. Ras , Adv. Mater. 2023, n/a, 2300306. 10.1002/adma.202300306 37052177

[49] W. S. Y. Wong , P. Bista , X. Li , L. Veith , A. Sharifi‐Aghili , S. A. L. Weber , H.‐J. Butt , Langmuir 2022, 38, 6224. 35500291 10.1021/acs.langmuir.2c00941 PMC9118544

[50] D. F. James , J Fluid Mech 1974, 63, 657.

[51] B. V. Derjaguin , Dok. Akad. Nauk SSSR 1946, 51, 517.

[52] W. S. Y. Wong , T. P. Corrales , A. Naga , P. Baumli , A. Kaltbeitzel , M. Kappl , P. Papadopoulos , D. Vollmer , H.‐J. Butt , ACS Nano 2020, 14, 3836. 32096971 10.1021/acsnano.9b08211 PMC7307963

[53] H. Sojoudi , S. Kim , H. Zhao , R. K. Annavarapu , D. Mariappan , A. J. Hart , G. H. McKinley , K. K. Gleason , ACS Appl. Mater. Interfaces 2017, 9, 43287. 29131948 10.1021/acsami.7b13713

[54] S. Huang , J. Li , L. Chen , X. Tian , J Phys Chem Lett 2021, 12, 3577. 33819039 10.1021/acs.jpclett.1c00760

[55] J. Zhang , S. Seeger , Angew. Chem., Int. Ed. 2011, 50, 6652. 10.1002/anie.201101008 21648031

[56] X. Deng , L. Mammen , H.‐J. Butt , D. Vollmer , Science 2012, 335, 67. 22144464 10.1126/science.1207115

[57] N. Gao , F. Geyer , D. W. Pilat , S. Wooh , D. Vollmer , H.‐J. Butt , R. Berger , Nat. Phys. 2018, 14, 191.

[58] A. Z. Stetten , D. S. Golovko , S. A. L. Weber , H.‐J. Butt , Soft Matter 2019, 15, 8667. 31528956 10.1039/c9sm01348b

[59] C. Ishino , M. Reyssat , E. Reyssat , K. Okumura , D. Quéré , EPL 2007, 79, 56005.

