# Supplementary material for: Designing Plastrons for Underwater Bubble Capture: From Model Microstructures to Stochastic Nanostructures
Source: Adv Sci (Weinh). 2024 Jul 2;11(33):2403366. doi: 10.1002/advs.202403366 (PMC11434225; doi:10.1002/advs.202403366)
Supplement: Supplementary file 1 — Supporting Information [file ADVS-11-2403366-s014.docx]

Supplementary Materials for

**Designing Plastrons for Underwater Bubble Capture:**

**From Model Microstructures to Stochastic Nanostructures**

William S. Y. Wong^1^*, Abhinav Naga^2,3^, Tobias Armstrong^4^, Bhuvaneshwari Karunakaran^1^, Dimos Poulikakos^5^, and Robin H. A. Ras^1^

Department of Applied Physics, School of Science,

Aalto University, FI-02150 Espoo, Finland^1^

Department of Physics,

Durham University, DH1 3LE Durham, United Kingdom^2^

Institute for Multiscale Thermofluids, School of Engineering,

University of Edinburgh, Edinburgh EH9 3FD, United Kingdom^3^

Laboratory for Multiphase Thermofluidics and Surface Nanoengineering

Department of Mechanical and Process Engineering,

ETH Zurich, Zurich, Switzerland^4^

Laboratory of Thermodynamics in Emerging Technologies,

Department of Mechanical and Process Engineering,

ETH Zurich, Zurich, Switzerland^5^

Keywords: Bubble Coalescence, Bubble Absorption, Bubble Rupture, Superhydrophobic, Super liquid repellent

*corresponding author: william.wong@aalto.fi

**This file includes:**

Experimental Section

Synthesis of Surfaces

- Synthesis of Model Micropillar Structures
- Chemical Vapor Deposition on Micropillars (Hydrocarbon, Silicone, and Perfluoroalkyl)
- Spray-Coating and Functionalization of Nanoparticulate Surfaces

Characterization of Surfaces

- High Speed Wetting Dynamics (Side View of Bubble Rupture)
- Ultra-High-Speed Wetting Dynamics (Top-Down View of Bubble Rupture)

Supplementary Discussion

- Analytical vs. Numerical Approximations, Stefan-Reynolds / Stokes-Reynolds
- Description of Plastrons and the Drainage of Hemi-Bubbles
- Inertial *vs.* Viscous Drainage – Blake Number
- Micro *vs.* Nanostructuring: Multiple Bubble Rupture-and-Absorption
- Energy Dissipation of the Moving Contact Line
- MATLAB Grid Array and Contact Line Computation
- Additional Governing Mechanism: Surface Chemistry

Supporting Figures S1-16

Supporting Movies M1-13

- 1) Failure of the Cassie State at High Gas Fraction, $\alpha$ = 97.5%.
- 2) Fixed Bubble (Wenzel vs Cassie) at $w$ = 125 µm and $\alpha$ = 90%.
- 3) Plastron Drainage (20 µm vs 125 µm at 90% $\alpha$).
- 4) Hemi-Bubble Formation with Small (1.2 cm^2^) vs Large (1.6 cm^2^) Grid.
- 5) Plastron Drainage (20um) with variable $\alpha$.
- 6) Split-Grid Analysis: $\alpha$ = 90% *vs.* 50% ($w$ = 20 µm). Bubble in Water.
- 7) Split-Grid Analysis: $\alpha$ = 90% *vs.* 50% ($w$ = 20 µm). Drop-Bubble in Air-Water.
- 8) Split-Grid Analysis: $\alpha$ = 90% *vs.* 50% ($w$ = 20 µm). Hexane in Water.
- 9) Graduated Gradient Grid: $s$ < 10 µm to $s$ = 100 µm, Δ = 0.25 µm. Bubble in Water.
- 10) Free vs Fixed Bubble ($w$ = 125 µm at 90% $\alpha$).
- 11) Algorithmic Bubble Tracking – MATLAB.
- 12) Fractional Contact Line Analysis (20 um width pillars with 43 um spacing).
- 13) Fractional Contact Line Analysis (Fumed Nanoparticles, Confocal).

**Experimental Section**

**Synthesis of Surfaces**

*Synthesis of Model Micropillar Structures*

Model micropillar arrays were designed in KLayout as negatives molds (photoresists forming pits, as .gds files) before fabrication *via* maskless lithography (MLA) methods in a cleanroom (Micronova, Aalto University). 4-inch silicon wafers (J14125, Siegert Wafer, <100>) were used as the substrate, with 10 mL of SU8-50 (Microchem) as the photoresist. Silicon wafers were first heated in a clean, dry oven at 120°C for 2 hours for dehydration, before spincoating SU-8-50 at 500 RPM for 5 s (Ramp: 200 RPM/s) and 1500 RPM for 30 s (Ramp: 300 RPM/s). Wafers were then pre-baked (Programmable hotplate, RHS) at 65°C for 15 minutes (Ramp: 21.6°C/min) and 95°C for 15 minutes (Ramp: 10°C/min). Baked wafers are then cooled down and loaded onto the MLA 150 (Heidelberg Instruments) and exposed using an optimal setting of -17 defocus with 300 mJ/cm^2^ (λ = 375 nm). Exposed wafers are then post-baked at 95°C for 12 minutes (Ramp: 19°C/min) and cooled at 3.75°C/min to room temperature. Wafers are then cooled down and immersed in a bath of developer solution for 20 minutes with swirling at 5-minute intervals. Wafers are then retrieved and washed using isopropanol and dried with a nitrogen air gun. A reactive ion etching (RIE) program (Oxford Instruments, Plasma RF generator) is then used to deposit a thin layer (30 nm) of fluoropolymer using CHF_3_ at 99.5 cm^3^/min over 10 minutes with a DC bias of 80V (Chamber pressure at 250 mTorr). Negative resist coated wafers are then retrieved from the cleanroom and templated using polydimethylsiloxane (PDMS, Sylgard 184). PDMS was prepared using cross-linked Sylgard 184 PDMS, mixed at a 1:10 weight ratio (1:10 g) of cross-linker-to-vinyldimethylsiloxane, respectively, in a 100 mL cup, stirred vigorously before evacuation (de-gas) in a clean desiccator to remove bubbles. Approximately 40 mL is poured onto a 4-inch wafer in petri dish (20 cm diameter) before curing in an oven at 80°C for 3h. The soft templated PDMS is then cut with a scalpel and peeled off the negative mold. This combination of PDMS flexibility and surface functionalization readiness (hydroxyls) provides the greatest experimental reach for our multi-parameter investigation.

*Chemical Vapor Deposition on Micropillars (Hydrocarbon, Silicone, and Perfluoroalkyl)*

To modify the PDMS micropillars, a series of silanes were used as functionalization agents, *via* chemical vapor deposition (Figure S12a). Octyltrichlorosilane (OTS, 97%, Sigma Aldrich) for hydrocarbon, dichlorodimethylsilane (DCDMS, ≥ 99.5%, Sigma Aldrich) for silicone, and 1*H*,1*H*,2*H*,2*H*-perfluorooctyltrichlorosilane (PFOTS, 97%, Sigma Aldrich) for perfluoroalkyl. The focus of this project relies primarily on the use of PFOTS as a standard. To functionalize these micropillars, they are first activated. To activate these substrates, they were oxygen-plasma treated for 10 minutes, at 100% power (Diener Electronic, PCCE, 300W). Activated surfaces were placed into a desiccator (20 cm diameter, $V$ = 4.2 L) at *ca.* 8 cm from the center, where 0.15 mL of silane (OTS, DCDMS, or PFOTS) was deposited, at *ca.* 2 cm lower than the glass substrates. The desiccator was then evacuated to 50 mbar. To create the thin functional layer, a 10-minute run time was executed before the functionalized surfaces were retrieved. Hydrophobic wetting of control glass slides (silica-based) in the same reaction chamber were used to confirm success of functionalization. After this, all functionalized surfaces were then evacuated at 50 mbar (*in situ* without silane present) for 30 min to remove residual silanes. A separate desiccator was used for each chemical (OTS, DCDMS, or PFOTS) to avoid cross-contamination. Functionalized surfaces were left to equilibrate with the ambient air environment (T = 20 °C, humidity = 30-70 %) for at least 1 day before testing.

*Spray-Coating and Functionalization of Nanoparticulate Surfaces*

Powdered silica SiO_2_ (Sigma Aldrich, fumed, 7 nm) in acetone suspensions (10 mg mL^-1^) were sprayed onto substrates at 3 bars with a flow rate of 0.2 mL s^-1^ from a 10 cm working distance using an air brush (nozzle diameter, 0.3 mm). 10 mL of the suspension was sprayed a dimensional area of 5 cm by 10 cm. A traverse rate of ca. 10 cm s^-1^ was maintained using guide rails on a custom-built spray rig. All surfaces were left to equilibrate with the ambient air environment (T = 20 °C, humidity = 30-70 %) for at least 1 day. To modify the silica (SiO_2_) nanoparticulate surfaces, *1H*,*1H*,*2H*,*2H*-perfluorooctyltrichlorosilane (PFOTS, 97%, Sigma Aldrich) was used as the functionalization agent, *via* chemical vapor deposition. As-deposited plain silica (SiO_2_) surfaces were placed into a desiccator (20 cm diameter, $V$ = 4.2 L) at *ca.* 8 cm from the center, where 0.5 mL of the functionalizing agent (PFOTS) was deposited, *ca.* 2 cm lower than the glass substrates. Silanes were deposited into the desiccator before evacuation to 50 mbar and kept for 30 minutes. After this, functionalized surfaces were then evacuated at 50 mbar (*in situ* without silane present) for 30 min to remove residual silanes. Functionalized surfaces were left to equilibrate with the ambient air environment (T = 20 °C, humidity = 30-70 %) for at least 1 day before testing.

**Characterization of Surfaces**

*High Speed Wetting Dynamics (Side View of Bubble Rupture)*

Surfaces synthesized using vapor deposition were assessed under-water (*ca.* 10 mm below the water line) for bubble rupture and contact dynamics (Figure S4b-d) using high speed camera imaging (Phantom v1610, U.S.A). A macro lens (Canon MP-E 65mm Macro) was used at *ca.* 2.5 X magnification. Rupture events are captured at 16,000 fps. The analysis of events is assisted *via* moving averages of 15 frames, giving an effective temporal resolution of 0.94 ms. Bubble rupture was performed only in liquid baths of milliQ water (18.2 MΩ.cm) to simplify descriptions. The water baths were changed every 2 hours. Bubbles were dispensed at a rate of 1 µL/s before natural detachment.

Bubble-rupture analysis with micropillars can be performed using both fixed bubble and/or free bubble configurations. In the fixed bubble configuration (Figure S4e, left panels), a bubble is inflated into plastron contact before rupture-induced detachment from the needle. In the free bubble configuration, a bubble detaches (from a needle) and floats freely up through the liquid bulk (*ca.* 1 mm) before encountering the plastron. Using a 19G needle, the needle-to-surface distance defines the testing mode, with 4.2 mm and 5.5 mm defining the fixed and free bubble configurations, at a far-field contact velocity of *ca.* 25-30 mm/s respectively. For the sake of conciseness, we will limit the scope of this study to bubbles contacting within this representative velocity profile (10^-2^ to 10^-1^ m/s). Significantly slower contact velocities beyond an order of magnitude (10^-4^ to 10^-3^ m/s) are known to result in range of 1-15 ms variations in absolute rupture time but should not influence trends observed. In *Phys. Rev. Lett.* **2019**, 122, 194501 (Figure 3), a variation in approach velocities from 0.5 to 2.8 mm/s changes film thinning-to-rupture time from a maximum of 15 ms down to 1 ms.^[^[^1^](#_ENREF_1)^]^ In both of our experimental cases, contact velocities are sufficiently high (25-30 mm/s) such that any influence on rupture time is likely much smaller than 1 ms (far below the intended parameter design space). Nonetheless, macroscopic deformations of bubbles are expected to be present, which will be consistently similar across experiments (see brief discussion below).

Therefore, a rapid rupture behavior (5-10 ms) will not be affected by contact velocity as rupture occurs almost immediately upon contact. For a slower rupture behavior (20 ms to 200 ms), bubbles are already at rest (contact area is constant, despite an oscillating center-mass profile). Bubble rupture is largely expected to occur at the contact rim of a dimpled bubble, contrasting the use of much slower approach velocities (*i.e.* 5 µm/s) where a nearly flat^[^[^2^](#_ENREF_2)^,^ [^3^](#_ENREF_3)^,^ [^4^](#_ENREF_4)^]^ film forms. As bubbles reach the surface, they slow down to a minimum velocity, registering bubble contact $t_{0}$ (Figure S4b,d). After a finite period of contact, the bubble ruptures and is absorbed into the surface (Figure S4b,d). This results in a sudden increase in the center-mass velocity, $v$, registering bubble rupture $t_{f}$. The time delay ($t_{f}$ – $t_{0}$) represents the rupture time measured (Figure S4b-e). The detection limit of this technique is *ca.* 5 ms (*i.e.* differences in rupture times below 5 ms cannot be discerned).

Due to the stochastic nature of film rupture, both rapid and slow bubble rupture can occur for a single surface. The statistical spread defines the differences between surface variants. 50 measurements are typically made over 3 cross-batch samples and represented as a scatter plot for reference. For rapid rupture timings (≤ 10 ms), fixed or free bubble configurations result in identical behaviors due to the almost-immediate rupture upon contact (Figure S4e, top panels, green circles). For slow rupture timings (≥ 50 ms), the fixed bubble configuration is more precise, as bubble contact is held in place by the needle. In contrast, the free bubble configuration (Figure S4e, bottom panels, red circles) can experience up to one bounce, causing a brief lift-off before contact is re-established with the surface. This leads to a larger (20 ms, black dotted line) error in measurements. Bubble rupture measurements are performed with the fixed-bubble configuration unless bubble absorption behavior is poor. In these instances, bubble rupture is often slow (> 50 ms) and the free bubble release technique can be used as an alternative. At *w* of 60-125 µm, surfaces experience poor post-rupture bubble absorption (tendency of Wenzel-wetting), resulting in poorer algorithmic detection by center-mass velocity as needle detachment may not occur. Therefore, rupture behavior was analyzed using the free-bubble configuration. On closer observation, the contact for these variants is often maintained even throughout the first full-contact bounce (Movie M10). Nonetheless, in the worst-case scenario: errors induced in damping and potential bubble lift-off may range up to 20 ms. These errors fall well within the standard errors (from stochasticity) computed for the domain ($w$ = 60-125 µm). Notably, surfaces with rapid bubble rupture properties (< 50 ms) are not affected by free/fixed bubble configurations as bubbles rupture within the first damping half cycle (~ 10 ms). A representative experimental video is included in Movie M1 for reference. Automatized algorithmic processing (using MATLAB) of bubble rupture analysis is included in Movie M11. Due to the deterministic nature of bubble absorption, measurements are performed with the free-bubble configuration to minimize the influence of the needle. Only post-rupture analysis (contact line spreading) is captured in these experiments.

*Ultra-High-Speed Wetting Dynamics (Top-Down View of Bubble Rupture)*

To track the initial location of bubble rupture, top-down through-surface imaging was performed using ultra-high-speed optical microscopy (Nikon Eclipse Ti2) with a 20X magnification objective (Nikon PLAN APO, 0.75 NA). The optical microscope was paired with a high-speed camera (Photron NOVA, S16). Rupture events were captured at 100,000 fps, with a shutter speed of 5 µs. A captive bubble (*ca.* 600 ± 150 µm in diameter, $n$ = 5) on a perfluoroalkyl-functionalized cover slip was moved down at *ca.* 10 µm/s towards micropillars ($\alpha$ = 90%, $w$ = 20 µm) in a bath (15 mL water) to achieve bubble rupture at the apex. 20 repeats were performed to confirm the occurrence of single-pillar solid-induced bubble film rupture. At 100,000 fps, the temporal resolution was sufficient in capturing the initial circular propagation wave culminating from the rupture event before encountering the outer micropillars. This enables accurate back-tracing of the origin of rupture *via* circle-fittting.

**Supplementary Discussion**

*Analytical vs. Numerical Approximations (Stefan-Reynolds / Stokes-Reynolds)*

We first consider the case where the location of rupture is on one of the contacting micropillars. This occurs, likely at the deformed rim of a contacting bubble. The millimetrically deformed contacting rim (dimple) will likely be of very similar area in all experimental cases due to the original bubble approach speed (20-30 mm/s). At this rim, numerous micropillars would have established contact, forming thin films that begin drainage. However, due to the 2D distribution present, drainage behavior becomes competitive, *i.e.* drainage of liquid from the top of one micropillar can flow towards another (*i.e.* crossflows). As a result, we observe the stochasticity present in our analysis. Eventually, a singular (or a few) micropillar induces the rupture event. We now consider this case with respect to a singular micropillar, alongside several key assumptions that follows the full numerical scheme defined by the Stokes-Reynolds model,

$\frac{\partial h_{f}}{\partial t}= \frac{1}{12\eta r}\frac{\partial}{\partial r}\left( r{h_{f}}^{3}\frac{\partial P}{\partial r} \right)$ (1)

where $r$ represents the local lateral dimension over which time ($t$)-dependent film drainage (height $h_{f}$) occurs.^[^[^5^](#_ENREF_5)^]^ $\eta$ represents the liquid viscosity. The pressure difference ($\frac{\partial P}{\partial r}$) includes local deformations described by the augmented Laplace pressure and interfacial forces, $\Pi\left( h_{f} \right) \Pi_{\mathrm{vdW}}+ \Pi_{\mathrm{EDL}} + \Pi_{\mathrm{st}}$ in which disjoining pressure, $\Pi\left( h_{f} \right)$, is the sum of van der Waals ($\Pi_{\mathrm{vdW}}$) dispersion forces, electrical double layer ($\Pi_{\mathrm{EDL}}$) forces, and steric ($\Pi_{\mathrm{st}}$) forces.^[^[^6^](#_ENREF_6)^]^ With the use of pure deionized water, the latter two contributions are negligible. Therefore, the pressure gradient is, for very thin films (< 100 nm) nearing rupture, primarily driven by disjoining pressure ($П_{vdW}=-\frac{A_{H}}{6\pi{h_{f}}^{3}}$). Under the Tabor-Winterton approximation,^[^[^6^](#_ENREF_6)^,^ [^7^](#_ENREF_7)^]^ the perfluoroalkyl-water-air (vacuum) system, $A_{H}^{FWG}$, has a destabilizing (film thinning) Hamaker constant of 1.51 x 10^-21^ J.

Firstly, the portion of the thin film that eventually ruptures on micropillars is very small. In fact, a uniform “on-pillar” rupture event does not occur even with our smallest micropillars (20 µm). The rupture event is located at different locations which varies alongside repeat experiments. From our direct observations using ultra-high-speed microscopy (see manuscript, Figure 2), the immediate location of rupture exists at a scale that is << 20 µm. Therefore, this suggests that the film thins unevenly across the tops of these micropillars, ultimately rupturing within a domain that is at 1 µm or less. To avoid the complication of cross-flows, we consider a single micropillar, with $r$ = 10 to 62.5 µm (with $w$ = 20 to 125 µm) since the geometry of the problem is 2D-symmetrical. At this scale, with an interfacing bubble at $R$ ≈ 2250 µm, significant curvatures are unlikely. However, the curvature (locations $i$ along $r$) can be approximated with,

$h_{i,f}= h_{0}-(\frac{r_{i}^{2}}{2R})$ (2)

which serves as an initial condition for the full solution to Equation 1. As a result of these $r$-dependent curvatures, we now have the Stokes-Reynolds-Young-Laplace (SRYL) model,

$\frac{\partial h_{f}}{\partial t}= \frac{1}{12\eta r}\frac{\partial}{\partial r}\left( r{h_{f}}^{3}\frac{\partial}{\partial r}(\frac{A_{H}}{6\pi{h_{f}}^{3}}-\frac{\gamma}{r}\frac{\partial}{\partial r}(r\frac{\partial h}{\partial r})) \right)$ (3)

In our experiments, $\Delta h_{f,max}=\frac{r^{2}}{2R}=$ 22 nm (at $r$ = 10 µm) up to 870 nm (at $r$ = 62.5 µm). In both boundary scenarios, the curvature is minimal but differ in orders of magnitude (both $\frac{\Delta h_{f,max}}{r}$≤ 0.015). The radii of curvature, $\kappa$, of these dimpled films is defined by bubble radius, $R$ ≈ 2250 µm, per how Equation 2 is defined.

At this juncture, if we assume the presence of a flat (or planar) film, where $\frac{\partial h_{f}}{\partial r}$ = 0, we arrive at the much more simplified analytical Stefan-Reynolds model,^[^[^4^](#_ENREF_4)^,^ [^5^](#_ENREF_5)^]^

$\frac{dh}{dt}= \frac{2\alpha h^{3}(\Delta P+ П_{vdW})}{3\eta r^{2}}$ (4)

where $\alpha$ is defined for a commonly accepted no-slip boundary condition (due to ease of interfacial contamination.^[^[^1^](#_ENREF_1)^]^ etc.) We also assume here that only vdW interactions are present, $\Pi\left( h_{f} \right) \Pi_{\mathrm{vdW}}$. For pure water, this is a reasonable assumption that also extends to the full numerical SRYL model. Both models often assume a similar starting and ending (point of rupture) film thickness.

The use of the Stefan-Reynolds model depends on the presence of a non-dimpled film, which is satisfied under conditions of very small film Capillary ($Ca$) numbers, at $Ca$ < ${10}^{-2}$,^[^[^4^](#_ENREF_4)^]^ where

$Ca= \frac{\eta Ur^{2}}{\gamma{h_{f}}^{2}}$ (5)

Where $\gamma$ is the liquid surface tension and $U$ is the characteristic velocity of drainage. This condition is not often satified, especially with a combination of large film radii, $r$ and high drainage velocity, $U$. In these cases, the Stefan-Reynolds model will only provide a qualitative guide.^[^[^4^](#_ENREF_4)^]^ Solution to the full numerical solution (SYRL) would improved estimates for the time-dependent variables ($h_{i,f} (t+j)$) and thus eventual rupture time ($t_{r}$).

To our knowledge, the closest numerical study in this domain was explored by Shah *et. al.*, whose work (*Phy. Rev. Fluids* 6, **2021**, 013603) investigated the rupture behavior of small radii films ($r$ ≈ 400 µm and below).^[^[^3^](#_ENREF_3)^]^ The full numerical computation, alongside a corresponding analytical simplication within our parameter space (*r* from 10 µm to 62.5 µm) was provided. Although Shah *et. al.* investigated the behavior of these thin films between two micrometric bubbles and not between a bubble and a rigid flat surface (micropillar), the only difference lies in a difference in the pre-factor of the surface tension contributions, with $\frac{\gamma}{2r}\frac{\partial}{\partial r}(r\frac{\partial h}{\partial r})$ instead of $\frac{\gamma}{r}\frac{\partial}{\partial r}(r\frac{\partial h}{\partial r})$. The absolute rupture time will be faster for the latter. However, this will not affect the scaling conclusions reached, where $t_{r}$ $\sim w^{1.1}$ (numerical) and $t_{r}$ $\sim w^{1.43}$ (analytical).

*Other Considerations, 1) Pillar Geometry:* In this work, we used square micropillars as the model geometry. For square pillars, only two planes (X-, and Y-) are in symmetry. In contrast to this, for round pillars, the film will be axisymmetric about the Z-axis. In both cases, the length scale that is of key interest, is the length scale ($r$) upon which the rupture occurs. In this case, the width represents that scale. For round pillars, the diameter (or radius) would represent that scale. Under consideration of scaling laws, if the width ≈ diameter, the same scaling^[^[^8^](#_ENREF_8)^,^ [^9^](#_ENREF_9)^,^ [^10^](#_ENREF_10)^]^ outcome (*i.e.* trend) is likely, which still heavily depends on the entire range of surfaces (20 µm to 125 µm). For the sake of reference, the largest corner-to-corner distance of a square pillar *vs.* the wall-to-wall width, varies by up to a factor of $\sqrt{2}$. This may create an offset (*i.e.* prefactor contributions), but not the overall outcome of the scaling (*e.g.* $t_{r}$ $\sim w^{1.41}$).

*Other Considerations, 2) Cross Flows:* In reality, an unknown contribution with bubble rupture on plastrons comes under the influence of crossflows (liquid flowing from one pillar to another), which may not be easy to computationally explore. For greater accuracy, a macroscopic non-axisymmetric model needs to be developed, considering a millimetric dimple, micrometric/nanometric film profiles, pillar axisymmetry (with or without corners), and the influence of crossflows (liquid flowing from one pillar to another). This falls beyond the scope of this experimental study but may be of future interest. As a result, while some intermediary conclusions may already be made, future work would have to focus on a non-axisymmetric domain with multiple features and their cross-interactions.

*Description of Plastrons and the Drainage of Hemi-Bubbles*

At 90% gas fraction (height of 80 µm, grid size of *ca.* 1.2 cm x 1.2 cm, total box volume ≈ 11.5 µL), the original volume is *ca.* 10.4 µL. At 50%, this volume is 5.75 µL. With an immersion depth of 1 cm, the pressure within the plastron, $P_{h}$ is *ca.* 98.1 Pa above atmospheric pressure. A bubble at *ca.* 4.5 mm in diameter has an initial volume of 47.7 µL. Bubble volume far exceeds available plastron volume (for microstructured surfaces).

After the bubble ruptures, the Laplace pressure is dynamic and changes over the course of absorption. Immediately after the bubble ruptures, the radius of curvature is low and $P_{L}$ is at its highest. However, the radius of curvature rapidly increases while $P_{L}$ rapidly decreases as the bubble takes the form of a hemi-bubble. This occurs spontaneously to achieve thermodynamic equilibrium. Note: The $P_{L}$ is not high even when considering a bubble with a radius of 2.25 mm. The maximum $P_{L}$ is thus bracketed at *ca.* 64.7 Pa and decreases from there. $P_{h}$ is always higher than $P_{L}$.

Table S1. Laplace Pressure per Equilibrium Hemi-Bubbles (Spherical Cap) at Different α

| Gas Fraction | a (mm) | h (mm) | r (m) | CA (°) | Laplace Pressure (Pa) |
| --- | --- | --- | --- | --- | --- |
| 50 | 0.0036 | 0.00196 | 0.00421 | 59 | 34.6 |
| 60 | 0.0034 | 0.00180 | 0.00412 | 53 | 35.3 |
| 70 | 0.0038 | 0.00149 | 0.00554 | 42 | 26.3 |
| 80 | 0.0039 | 0.00121 | 0.00682 | 38 | 21.3 |
| 90 | 0.0052 | 0.00080 | 0.01700 | 22 | 8.6 |

The sphericity ($Sp$) and the radius of curvature ($r_{c}$, *via* the spherical cap equation) at equilibrium (Figure S12) can be evaluated for experiments with gas fraction ($\alpha$ = 50-90%) variation. $Sp= \frac{l_{x}}{l_{y}}$ is defined by the lateral length, $l_{x}$ and vertical length, $l_{y}$ of the hemi-bubble. If the plastron is connected to the atmosphere by needle puncture (26G), the spontaneously formed hemi-bubbles can be drained (see manuscript, Figure 3q) within timescales at the order of 1-2s ($\alpha$ = 90%) to 20 ± 5 s ($\alpha$ = 50%), See Movie M5. The initial volume profile of each hemi-bubble indicates that a larger gas fraction, $\alpha$ can lead to the immediate absorption of almost 100% more air (within 50 ms) before gradually slower drainage. The slower drainage is dominated by the viscous effect, which is initially surprising because the drainage medium is air. However, a computation of the average nondimensional Blake number (generalized Reynolds number through porous media, see below) during initial absorption: 0.1 to 1.5 s (inertial *vs.* viscous effects), $B= \frac{u\rho D_{h}}{\mu(1-\alpha)}$, gives an average of *ca.* 1.13 x 10^-1^ ($\alpha$ = 90%) to 3.95 x 10^-4^ ($\alpha$ = 50%). $u$ is the flow velocity, $\rho$ and $\mu$ is the density and dynamic viscosity of air, and $D_{h}= \frac{4A}{P}$ is the hydraulic diameter, where $A$ is the cross-sectional flow, and $P$ is the wetted perimeter of the cross-section. As $B$ is << 1, particularly with decreasing gas fraction, drainage is predominated by viscous effects. Detailed calculations are included below. The drainage first occurs with pinned contact lines (bubble CA recedes) before eventual depinning, driven primarily by hydrostatic pressure. However, these timescales are much longer than the time taken to establish the bubble profiles (typically within 50 ms).

Table S2. Blake number of initial bubble absorption ($t$ = 0.1 to 1.5 s, average) with variable $\alpha$

| Gas Fraction  $\alpha$ (%) | Flow Velocity (m/s) | Hydraulic Diameter (m) | Ave. Blake Number (-) |
| --- | --- | --- | --- |
| 50 | 1.40 x 10^-2^ | 2.00 x 10^-5^ | 3.95 x 10^-4^ |
| 60 | 1.65 x 10^-4^ | 1.20 x 10^-5^ | 3.59 x 10^-4^ |
| 70 | 3.30 x 10^-4^ | 4.66 x 10^-5^ | 3.66 x 10^-3^ |
| 80 | 2.15 x 10^-4^ | 7.99 x 10^-5^ | 6.14 x 10^-3^ |
| 90 | 5.82 x 10^-5^ | 1.79 x 10^-4^ | 1.13 x 10^-1^ |

*Inertial vs. Viscous Drainage – Blake Number*

Drainage of a hemi-bubble after a direct plastron-to-atmosphere connection is slower with hemi-bubbles having lower curvatures ($\alpha$ = 50%, 10-20 s vs. $\alpha$ = 90%, 1-2 s). Therefore, the phenomenon is likely not dictated by Laplace pressure. Hydrostatic pressure represents the primary driving force, but the nature of the different drainage behaviors remains intriguing. Here, we use the nondimensional Blake number, $B$, which accounts for inertial *vs.* viscous effects of flow in porous media (generalized Reynolds number) to explain why drainage is occurring under such contrasting differences.

$B= \frac{u\rho D_{h}}{\mu(1-\alpha)}$ (6)

where $u$ is the flow velocity, $\rho$ and $\mu$ is the density and dynamic viscosity of air, and $D_{h}= \frac{4A}{P}$ is the hydraulic diameter, where $A$ is the area of the cross-sectional flow, and $P$ is the wetted perimeter of the cross-section.

Firstly, the unit square of each surface is known, which is made up of two half pillars’ widths (see manuscript, Figure 1), $w$ and spacing, $s$, giving the unit square’s area,

$A_{unitsquare}={(s+w)}^{2}$ (7)

Accounting for the gas fraction, $\alpha$, the area of cross-sectional flow,

$A= {\alpha\times A}_{unitsquare}$ (8)

Based on the unit square, the wetted perimeter of the cross-section,

$P=8 \times\frac{w}{2}$ (9)

With $A$ and $P$, $D_{h}$ can be computed. Next, the flow velocity, $u$, can be experimentally determined through the rate of deflation $\frac{dV}{dt}$ of bubble volume, $V$ (graphically extracted), over time, $t$. The spherical cap’s contact radius ($a$) is graphically extracted at each time step during drainage. Using an axisymmetric assumption of the bubble contact area,

$A_{bubble}= \pi a^{2}$ (10)

Under this bubble, the unit squares, $n$, in contact with the bubble can be determined,

$n= \frac{A_{bubble}}{A_{unitsquare}}$ (11)

Using the number of unit squares, $n$, the total area for cross-sectional flow is determined,

$A_{total}= n\times$ $A$ (12)

With the total available flow area, the flow velocity, $u$, can then be approximated,

$u\approx(\frac{dV}{dt}$)/$A_{total}$ (13)

Considering that datasets are acquired at 500 fps, each time interval is approximately 0.002 s. Therefore, a moving-average of 100 data points is used to reduce noise, *i.e.* at every 0.2 s. This data extraction domain is a good approximation due to the linear nature of the drainage between $t=0.1 to 1.5 s$. Within this time domain, an average Blake number of *ca.* 1.13 x 10^-1^ ($\alpha$ = 90%) to 3.95 x 10^-4^ ($\alpha$ = 50%) was computed. This is highly indicative of an increasingly viscous regime through which hemi-bubbles drain under decreasing $\alpha$.

*Micro vs. Nanostructuring: Multiple Bubble Rupture-and-Absorption*

Microstructured surfaces are indeed capable of achieving fast (*ca.* 20 ms) bubble rupture. However, they cannot effectively absorb bubbles within the same timescale (1-20 s). The formation of pinned hemi-bubbles highlights a useability problem. This becomes particularly evident during rapid and continuous multi-bubble interactions.

*Microstructured Surfaces:* The landing and rupturing of the first bubble causes the immediate locality to be defunct due to hemi-bubble formation (Figure S13a-b). The presence of the hemi-bubble prevents the rupture of a second or third incoming bubble until it is drained (Figure S13a, c-d). Even under continuous drainage by a connection to the atmosphere, an absorption time in the order of approximately 1 s to 20 s is required, which is 10^1^ to 10^3^ times longer than the actual film rupture event (*ca.* 50 ms, Movie M5). This bottleneck hinders continuous bubble capture operation in many engineering scenarios (*i.e.* foams,^[^[^2^](#_ENREF_2)^,^ [^8^](#_ENREF_8)^]^ flotation,^[^[^9^](#_ENREF_9)^,^ [^10^](#_ENREF_10)^]^ catalytic evolution^[^[^11^](#_ENREF_11)^,^ [^12^](#_ENREF_12)^]^ etc.) where multiple bubble-to-surface interactions occur quickly. When incoming bubbles land on the undrained hemi-bubble, they bounce on the soft interface, before coming to rest. The eventual bubble-to-bubble coalescence occurs at *ca.* 5 s after initial contact (Figure S13c,d).

*Hierarchical Nanostructured Surfaces:* With a hierarchical nanostructured surface, bubble capture behavior becomes well-defined even across multi-bubble interactions. Rupture of the first bubble (Figure S13e-f) leads to a slightly inflated plastron while the following bubbles (second or third or more) continue to be rapidly ruptured within the timescale of the first bubble (Figure S13e, g-h). The quantified nature of rupture, *via* sphericity, $Sp$, analysis of the multiple tracked bubbles (No. 1-3) for microstructured and hierarchical nanostructured surfaces is included in Figure S14 for reference.

As discussed in the fractional contact line analysis (see manuscript, Figure 4), significant differences in fractional contact lines exist between microstructured surfaces (Figure S13i) *vs.* hierarchical nanostructured surfaces (Figure S13j) during the bubble absorption step. The hierarchical (*i.e.* unevenly heighted) nature of the latter leads to a 1) loosely-bounded contact line while the 2) unevenness of the interface places continued pressure on incoming bubbles. Unlike the microstructured plastron whose interface is nearly perfectly flat (Figure S13i, black arrows), the overall unevenness of the hierarchical nanostructured plastron maintains point locations (Figure S13j, black arrows) where rupture events can occur. Note: the hierarchical nanostructured plastron will also begin to fail once it is sufficiently gas-flooded, resulting in bubble-to-bubble rupture timescales. However, due to surface hierarchy, this requires a larger number of ruptured bubbles.

*Energy Dissipation of the Moving Contact Line (During Bubble Absorption Pre-Equilibrium)*

*1) Viscous Dissipation*

The contact line progresses at capillary wave velocities in all cases (up to *ca.* 1 m/s, Figure S8) which would result in viscous dissipation from the start ($t_{0}$) until equilibrium ($t_{eq}$)

$\dot{E_{v}}= \int_{0}^{t_{eq}} 2\pi r_{b}f\eta U^{2}dt$ (14)

where $\eta$ is the dynamic viscosity and $U$ is the contact line velocity.^[^[^13^](#_ENREF_13)^,^ [^14^](#_ENREF_14)^,^ [^15^](#_ENREF_15)^,^ [^16^](#_ENREF_16)^]^ A friction correction factor, $f$ is applied for different surfaces.^[^[^16^](#_ENREF_16)^]^ In our scenario, $f$ is a constant since similar surface chemistry is employed (perfluoroalkylated PDMS). $2\pi r_{b}$ represents the dynamically expanding circumferential contact line of the bubble, where $r_{b}$ is the hemi-bubble radius. If we assume that contact with the gas domains possesses a negligible contribution, $2\pi r_{b}$ may be more accurately represented by $C_{f}$, the instantaneous length of the bubble circumference in solid contact, so-termed effective contact line length.

$E_{v}= \int_{0}^{t_{eq}} C_{f}f\eta U^{2}dt$ (15)

The upper limit for viscous dissipation may be estimated by using maximum $C_{f}$ (*i.e.* ${C_{f}}^{max}$) integrated with the maximum time taken for the contact line to stabilize (*ca.* 10 to 50 ms).

*2) Depinning-Induced Dissipation*

As the contact line moves over the micropillars, it experiences additional dissipation induced by micropillar-induced pinning and capillary bridge rupture. The outcome of the latter effect results in the visual artifact of remnant microdroplets (Main manuscript, Figure 2b). Following previously developed formalism by Butt *et. al*, ^[^[^17^](#_ENREF_17)^]^ James^[^[^18^](#_ENREF_18)^]^ and Derjaguin^[^[^19^](#_ENREF_19)^]^, we let the contact line pinning force exerted by each micropillar be $F_{p}$. The force required to break a capillary bridge off the top of a square-shaped micropillar is defined here with a circumference-defined vertical force component,

$F_{p}=4w\gamma sin(\varphi)$ (16)

where $\varphi$ represents the complementary angle to $\theta_{rec}$, at ($180- \theta_{rec}$). $\theta_{rec}$ represents the (water) receding contact angle on each micropillar that pins the contact line. As the bubble spreads, water recedes in a local stick-slip progression, with work of adhesion being an integral of the force over capillary bridge extension,

$W_{p}= \int_{0}^{\delta_{c}} F_{p}$ $d\delta$ (17)

where $\delta_{c}$ is the maximum extension of a radially symmetric capillary bridge, with radius ≈ $\frac{w}{2}$.

The extension of the capillary bridge is approximated by,^[^[^18^](#_ENREF_18)^,^ [^19^](#_ENREF_19)^]^

$\delta=\frac{w}{2}sin\left( \varphi\right)\left\{ ln\left[ \frac{4\kappa}{\frac{w}{2}\left( 1+\cos\left( \varphi\right) \right)} \right]-0.5772 \right\}$ (18)


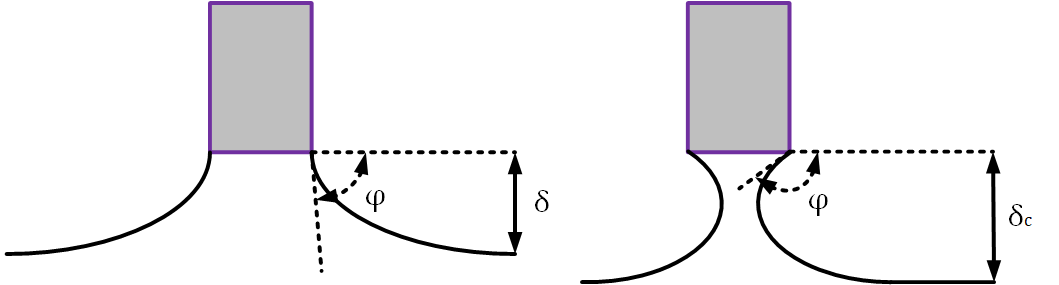


Schema 1. Schematic of Axisymmetric Capillary Bridge Rupture (Vertical Component).

Where $\kappa$ is the capillary length, $\kappa= \sqrt{\frac{\gamma}{\rho g}}$. For water, $\kappa$ = 0.002725 m at 20 °C. 0.5772 is the Euler constant. Equation 18 was originally defined under the condition where an extended liquid-air interface becomes flat at large distances. Therefore, it is valid when $\frac{2(s+w)}{w}\ll1$. At smaller $\alpha$, $\delta$ becomes an overestimate. As observed, $\delta\sim w$.^[^[^18^](#_ENREF_18)^]^ As we know that capillary bridge rupture would leave behind microdroplets (Main manuscript, Figure 2b), the condition of an insufficiently high receding contact angle, $\theta_{rec}$ is likely present,

$180^{\circ}- \varphi> \theta_{rec}$ (19)

$\theta_{rec}$ is typically around 90°^[^[^20^](#_ENREF_20)^,^ [^21^](#_ENREF_21)^]^, a reasonable estimate for the receding contact angle of perfluoroalkylated surfaces. For simplicity, we assume the maximum force, achieved would be at $\varphi= 90^{\circ}$during bridge rupture (using equation 18).

$\delta_{c}=\frac{w}{2}\left\{ ln\left[ \frac{8\kappa}{w} \right]-0.5772 \right\}$ (20)

If a lateral component (tilt) is present, as what is often observed in receding contact lines, the expression of $\delta_{c}$ is different, but $\delta_{c} \sim w$.^[^[^17^](#_ENREF_17)^]^ As a result, the work of adhesion by depinning, by combining equations 17, 18 and 20, $W_{p} \sim w^{2}$. We assume here that $W_{p}$ is a constant between different $\alpha$ as the same pillar topography is present (*i.e.* $w$). However, the number of micropillars that depin vary across different $\alpha.$ The energy dissipated within the hemi-bubble domain is therefore $E_{p}= \pi r_{b}^{2}nW_{p}$, where $n$ is the number of micropillars per unit area.

*Overall Dissipation by Moving Contact Line*

The combined energy dissipated as the contact line progresses across micropillars is $E= E_{p}+ \int\dot{E_{v}} dt$. Both contributions depend heavily on where the contact line currently resides and the solid surface that it contacts. The energy lost scales with the hemi-bubble radius ($r_{b}$), having a first-order dependency on the effective contact line length ($C_{f})$ and a second-order dependency on an area-effect: capillary bridge rupture ($nr_{b}^{2}$). In the former, only the solid contact line dissipation is considered as gas contact is negligible. In the latter, an integral of the total area multipled with $n$ under different $\alpha$should be considered.

*MATLAB Grid Array and Contact Line Computation*

Surfaces are generated in MATLAB with variable gas fraction, $\alpha$ and/or variable feature sizes, $w$ that were experimentally assessed. Thereafter, a bubble contact line is generated and expanded, defined by a radius ($r_{b})$ starting from 100 µm, progressing at 5 µm steps, until 10000 µm (Movie M12). As the contact line grows, the overlap between the bubble contact line and the simulated pillar tops are computed as the actual contact line length (and fraction, per Figure S9) with each step, $C_{f}$. This estimates the instantaneous solid-contact regardless of surface (in)homogeneity. For instance, if the contact points of the liquid-solid interface in a nanostructured surface is known, simulating the actual fractional contact line is also possible (Movie M13). This simulation is not time- nor step- size (Figure S15) dependent. The contact line length (and fraction) represents the only dependent variable from which we assess the increasing adhesion that eventually halts the spreading bubble. The simulation code is provided below for re-use and reference.

%% Square Array Generator and Contact Line Tracker %%

clc;clear

%% Simulation Initialization Parameters

% 20-43um 20-25um 20-17um 20-12um 20-8um

% 20-43um 40-86um 60-130um 80-173um 100-216um 125-270um

% Loop algo for Square array dimension (um)

sqsidevector = [20 20 20 20 20]; % Width of Squares

sqspacevector = [43 25 17 12 8]; % Space between Squares

for i = 1:numel(sqsidevector)

sqside = sqsidevector(i);

sqspace = sqspacevector(i);

% Initialize a grid of 1 cm x 1 cm (10000 um by 10000 um)

gridsize = 10000;

gridarray = zeros(gridsize);

% Define Initial Contact Dimension and Marching Length in um

Initialr = 100; % Radius in um

stepsize = 5; % Move contact line XX um at a time.

% Plot skipped frames to speed up simulation

counter = 5; % counter might be sensitive to output divergence - be careful

% Create Label for File

sqsidestr = int2str(sqside);

sqspacestr = int2str(sqspace);

gridsizestr = int2str(gridsize);

%% Simulation Space for Grids

% Initialize Figure to prevent screen hijack

fg = figure(1);

% Square Space

Squarenumber = floor(gridsize/(sqspace + sqside));

Subunit = round(sqspace+(sqside/2));

% Populate the grid with array dimensions

for x = 1:(Squarenumber-1)

for y = 1:(Squarenumber-1)

% Make each unit square in sequence

xshift = x*(sqspace+sqside);

yshift = y*(sqspace+sqside);

for j = xshift:(xshift+(sqside/2)) % x axis squares

for k = yshift:(yshift+(sqside/2)) % y axis squares

% Top Left Square

gridarray(j,k) = 1;

% Top Right Square

gridarray(j+Subunit,k) = 1;

% Bottom Left Square

gridarray(j,k+Subunit) = 1;

% Bottom Right Square

gridarray(j+Subunit,k+Subunit) = 1;

end

end

end

end

set(0,'CurrentFigure',fg);

set(fg,'Position', get(0, 'Screensize'));

subplot(2,2,1), imshow(gridarray)

xlabel(append(gridsizestr, ' um'))

ylabel(append(gridsizestr, ' um'))

title('Tracked Bubble on Grid Contact')

hold on

% Create a circle that grows - i.e. spreading contact line

% Preallocate circle grid

circlearray = zeros(gridsize);

circenter = [(gridsize/2), (gridsize/2)]; % both x and y axis

% Preallocate an overlap array to collect overlap

overlaparray = zeros(gridsize);

%% Circle Growth Simulation

X = 0; % Initialize frame counter

cumcontactlinelengthstore(1) = 0; % Initialize cumulative counter

% Grow circle using iteration of radius (in um)

for r = Initialr:stepsize:(gridsize/2)

% Total Contact Line Length (Air and Solid)

contactlinelengthtotal = 2*pi*r;

% Framestep of growth

X = X + 1;

% Store bubble size

rstore(X) = r;

% Stop simulation at end of grid

if r == (gridsize/2)

break;

else

end

% Map circle in x axis (do not switch on with y axis computation)

for circlex = (circenter(1)-r):(circenter(1)+r)

ycirclecoordfloor = floor(sqrt(r^2-(circlex-circenter(1))^2) + circenter(2));

ycirclecoordceil = ceil(sqrt(r^2-(circlex-circenter(1))^2) + circenter(2));

% Map the circle in X-axis with ones

circlearray(circlex,ycirclecoordfloor) = 1;

circlearray(circlex,ycirclecoordceil) = 1;

% Map the shift from the Bottom Arc

yshiftceil = ycirclecoordceil - circenter(1);

yshiftfloor = ycirclecoordfloor - circenter(1);

% Map the reflected circle

circlearray(circlex,circenter(1)-yshiftfloor) = 1;

circlearray(circlex,circenter(1)-yshiftceil) = 1;

end

clear circlex ycirclecoordfloor ycirclecoordceil yshiftfloor yshiftceil

% Plot

set(0,'CurrentFigure',fg);

set(fg,'Position', get(0, 'Screensize'));

subplot(2,2,2), imshow(circlearray)

xlabel(append(gridsizestr, ' um'))

ylabel(append(gridsizestr, ' um'))

title('Simulated Bubble')

pause(0.1)

% Compute the contact

% Overlap gridarray and circlearray

overlaparray = gridarray + circlearray;

contactpoints = find(overlaparray == 2);

contactlinelength = numel(contactpoints);

contactlinesolidfraction = (contactlinelength/contactlinelengthtotal)*100;

contactlinelengthstore(X) = contactlinelength;

cumcontactlinelengthstore(X+1) = contactlinelength + cumcontactlinelengthstore(X);

contactlinesolidfractionstore(X) = contactlinesolidfraction;

if rem(r,counter) == 0

% Plot contactpoints on grids with counter

% If contact points are present, save coordinates

[xcontact, ycontact] = find(overlaparray == 2);

set(0,'CurrentFigure',fg);

set(fg,'Position', get(0, 'Screensize'));

subplot(2,2,1), plot(xcontact,ycontact,'o');

hold on

% Plot Contact Line Length

set(0,'CurrentFigure',fg);

set(fg,'Position', get(0, 'Screensize'));

subplot(2,2,3), plot(r,contactlinelength,'o')

xlabel('Bubble Contact in Radius (r, um)')

ylabel ('Length of Contact Line (um)')

title(append(gridsizestr, ' um grid with ', sqsidestr, ' um width pillars with ', sqspacestr, ' um spacing'))

hold on

% Plot Contact Line Solid vs. Total Contact Line Fraction

set(0,'CurrentFigure',fg);

set(fg,'Position', get(0, 'Screensize'));

subplot(2,2,4), plot(r,contactlinesolidfraction,'o')

xlabel('Bubble Contact in Radius (r, um)')

ylabel ('Contact Line Solid Fraction (%)')

title(append(gridsizestr, ' um grid with ', sqsidestr, ' um width pillars with ', sqspacestr, ' um spacing'))

hold on

% Capture all frames

F(X) = getframe(gcf);

elseif rem(r,counter) ~= 0 % counter not in line with frame

F(X) = getframe(gcf); % Capture all frames without change

continue;

end

% Clean matrix

circlearray = zeros(gridsize);

overlaparray = zeros(gridsize);

clear contactlinelength contactpoints

end

%% Prepare video store

% Rename File to Parameters

filenamestr = append(gridsizestr, ' um grid with ', sqsidestr, ' um width pillars with ', sqspacestr, ' um spacing');

% Create the video writer with 1 fps

writerObj = VideoWriter(filenamestr);

% Set the seconds per image

writerObj.FrameRate = 60;

% Open the video writer

open(writerObj);

% Write the frames to the video

for i = 1:length(F)

% convert the image to a frame

frame = F(i);

writeVideo(writerObj, frame);

end

% close the writer object

close(writerObj);

%% Prepare excel readout

contactlinelengthstore = contactlinelengthstore';

contactlinesolidfractionstore = contactlinesolidfractionstore';

% Crop Initialization Point

cumcontactlinelengthstore = cumcontactlinelengthstore(2:end);

cumcontactlinelengthstore = cumcontactlinelengthstore';

rstore = rstore';

% Write for specific user file

filename = filenamestr;

% Remove warning

warning('off','MATLAB:xlswrite:AddSheet')

% Length data

xlswrite(filename,contactlinelengthstore,'CL Length (um)')

% Cumulative Length data

xlswrite(filename,cumcontactlinelengthstore,'Cum CL Length (um)')

% Fraction CL data

xlswrite(filename,contactlinesolidfractionstore,'Contact Line Fraction (%)')

% Bubble Radius data

xlswrite(filename,rstore,'Bubble Contact in Radius (um)')

%% Complete

close('all')

clear rstore contactlinesolidfractionstore contactlinelengthstore contactlinglength contactlinesolidfraction r cumcontactlinelengthstore

clear gridarray circlearray overlaparray

end

*Additional Governing Mechanism: Surface Chemistry*

A secondary question to plastron-induced bubble rupture discusses the influence of surface chemistry. As film thinning leading to bubble rupture is shown to be at least co-dominated by the nature of the solid chemistry, the degree of influence by different surface chemistries on disjoining pressure should be elaborated. As such, micropillars with 20 µm width at 80 µm height and wall-to-wall distance of 43 µm (Figure S16a) are used for surface treatment and characterization (Figure S16b). They are functionalized using hydrocarbon, octyltrichlorosilane (OTS, 97%, Sigma Aldrich); silicone, dichlorodimethylsilane (DCDMS, ≥ 99.5%, Sigma Aldrich); and perfluoroalkyl, *1H*,*1H*,*2H*,*2H*-perfluorooctyltrichlorosilane (PFOTS, 97%, Sigma Aldrich). Notably, with the use of pure milliQ water (18.2 MΩ.cm), none of the variants were able to significantly outperform the others (Figure S16c), with each bearing an average and statistical rupture timing of 10-20 ms with overlapping errors.

A closer inspection of the interaction forces through Hamaker constants (water as a medium) reveal an intriguing detail. Under the Tabor-Winterton approximation,^[^[^6^](#_ENREF_6)^,^ [^7^](#_ENREF_7)^]^ the perfluoroalkyl-water-air (vacuum) system, $A_{H}^{FWG}$, has a destabilizing (film thinning) Hamaker constant of 1.51 x 10^-21^ J. However, the hydrocarbon-water-air (vacuum) and silicone-water-air (vacuum) systems have stabilizing Hamaker constants of $A_{H}^{HWG}$, -1.25 x 10^-20^ J and $A_{H}^{SWG}$, -3.71 x 10^-21^ J respectively. This suggests that the latter variants should not be capable of bubble rupture if film rupture is completely dependent on solid surface chemistry. The disjoint between Hamaker constants and experimentally observable phenomena warrants further future investigation.

**Supporting Figures**


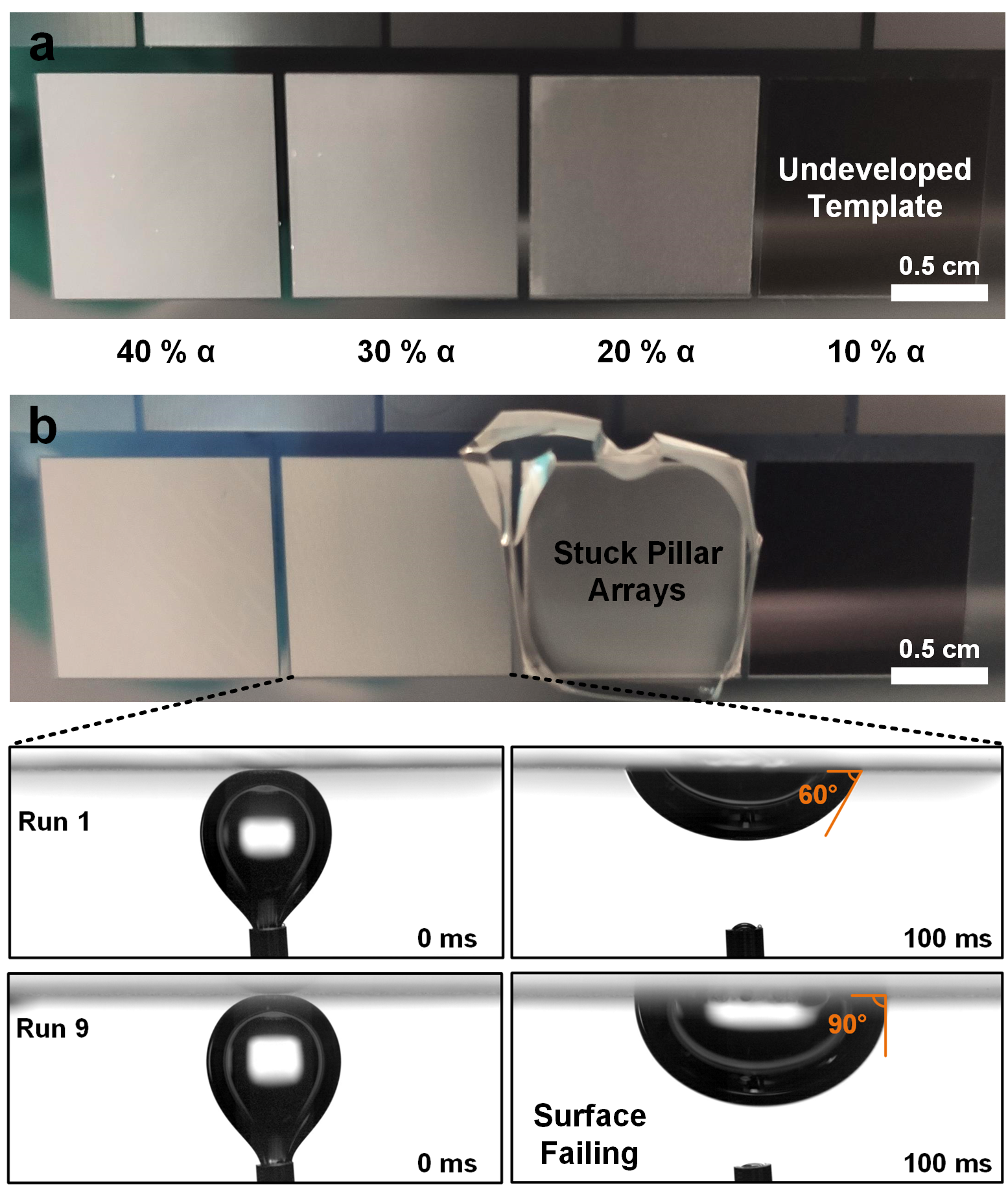


**Figure S1. Lithography-Templating-Assessment Lower Limit, at Gas Fraction, α = 20-30%.** a) Lithography was unable to develop templates at 10% α due to the extremely small interspatial distance of *ca.* 1 µm. b) While negatives at α = 20% can be fabricated, templating was not possible due to the extremely high adhesion between the positive PDMS templating and the SU-8 negatives. Positive PDMS templates at α = 30% can indeed be templated, but they suffer from rapid degradation of surface behavior after 5-10 runs of experimentation, with the macroscopic contact angles drastically changing – which is indicative of agglomerated pillars and locally altered gas fraction configurations.


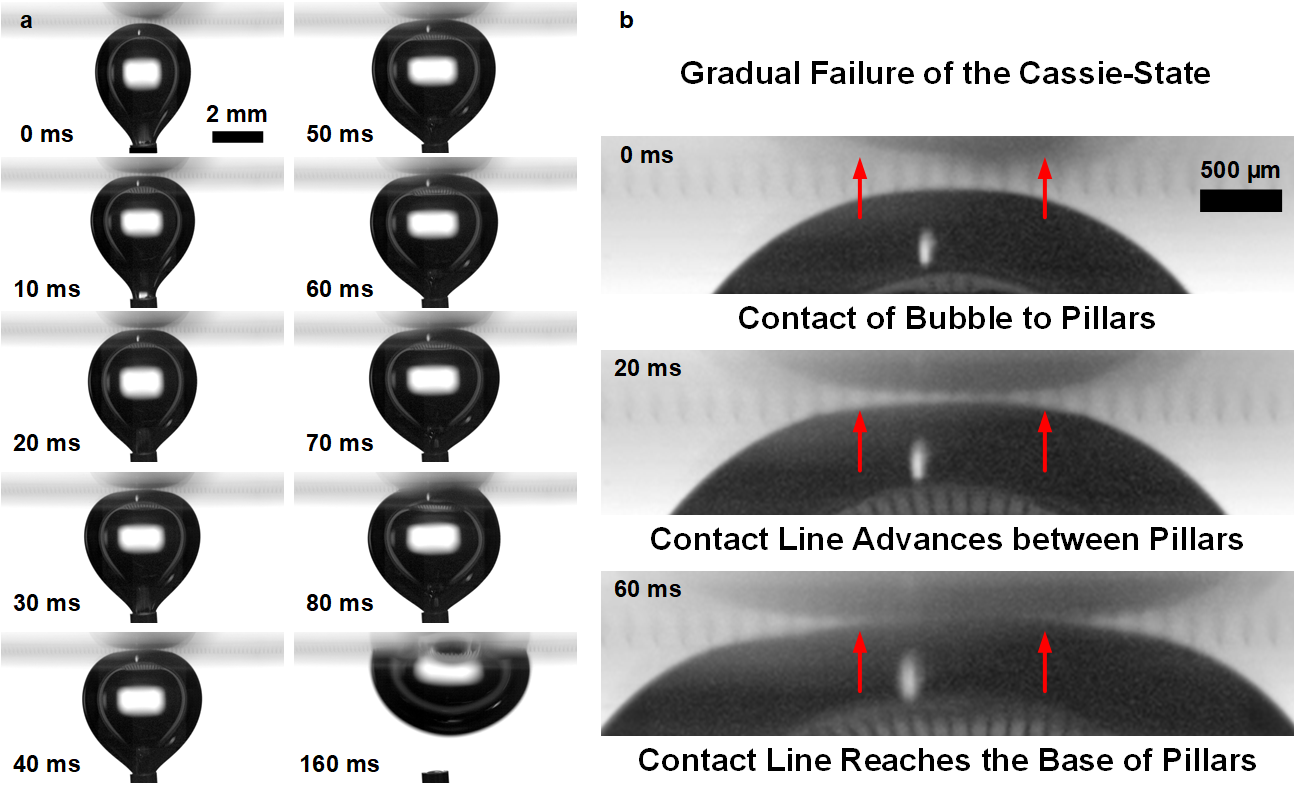


**Figure S2. Lithography-Templating-Assessment Upper Limit, at Gas Fraction, α = 97.5%.** a) The widely spaced pillars ($s$ = 106 µm) did not allow a stable Cassie-state, as the sandwiched b) water film gradually enters the gaps between the pillars. This hinders the actual rupture behavior, and for the sake of consistency in the main manuscript, will not be investigated in further details.


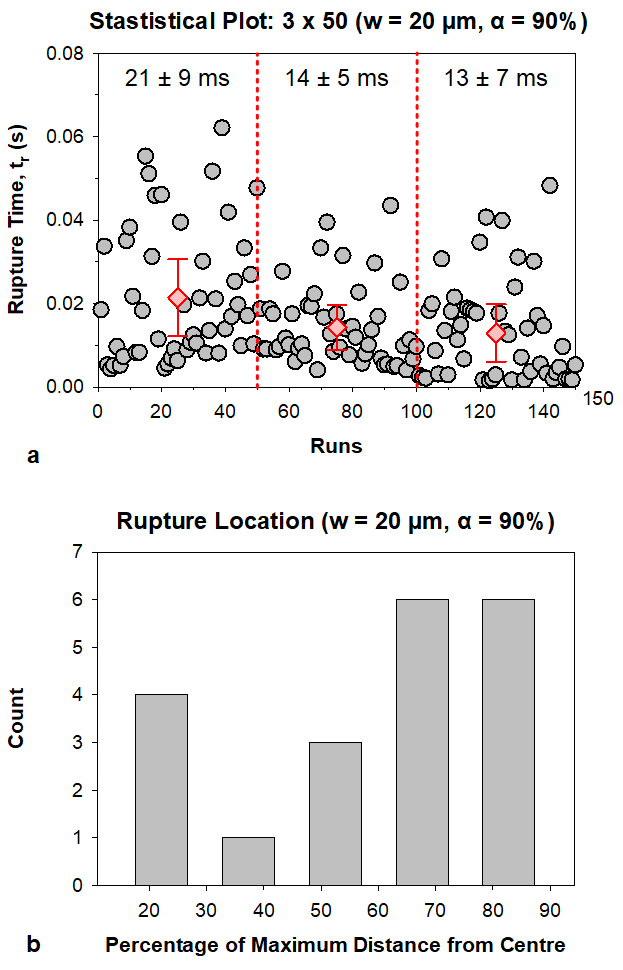


**Figure S3. Statistical Assessment of Repeat Experiments.** a) Bubble Rupture Time: Representative datasets from the most commonly used variant, $w$ = 20 µm, $\alpha$ = 90 %. 3 repeat batches of experiments (different synthesis and different days) were performed, with 50 datasets collected per repeat experiment using n = 3 samples each, *i.e.* 9 samples in total. The measured rupture times were clearly very similar, with overlapping statistical data spread and averaged values (with standard errors). b) Bubble Rupture Location: The distribution of bubble rupture location was assessed over n = 20 repeats, with rupture events all occurring on the solid micropillars. None occurred on the gas fraction. The percentage of maximum distance from the center extended from the middle of each pillar (0 %) to the edges of each pillar (71 %) until the corners of each pillar (100 %). In Figure S5, the raw data is presented for each experiment (n = 20) analyzed.

**
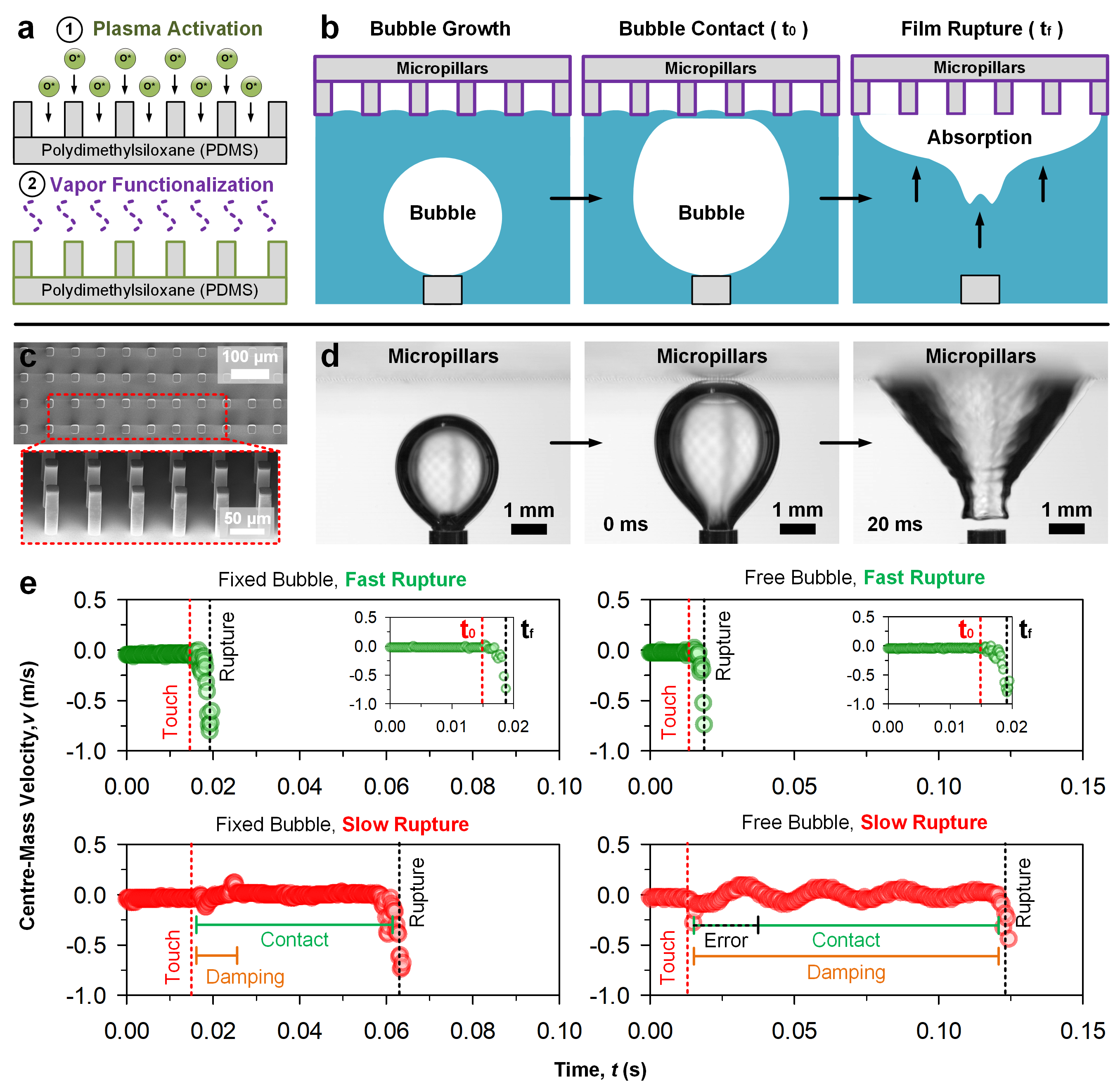
**

**Figure S4. Plastron-Induced Bubble Rupture: Synthesis and Characterization.** a) Synthesis of silane-functionalized superhydrophobic micropillars: 1) plasma-activation, 2) vapor-functionalization, and b) underwater characterization (milliQ, 18.2 MΩ.cm). With the use of clean milliQ water, the influence of $\Pi_{\mathrm{EDL}}$ and $\Pi_{\mathrm{st}}$ contributions are eliminated. c) Illustrative scanning electron micrograph of PDMS micropillars (fabricated from negative mold templating). d) Characterization of micropillars *via* fixed (on needle) bubble contact: Schematic in b) and experiment in d). e) The center mass velocity of bubbles is tracked, showing the behavior of fixed bubbles experiencing fast rupture (< 10 ms) (top left, green) or slow rupture (> 50 ms) (bottom left, red). When compared to free bubbles, fast rupture does not experience any detectable differences in rupture time (top right, green) while slow rupture may experience bubble-surface detachment (up to 20 ms) due to the first bounce cycle. Data presented is illustrative as the rupture phenomenon is statistical and only becomes representative with multiple repeats.


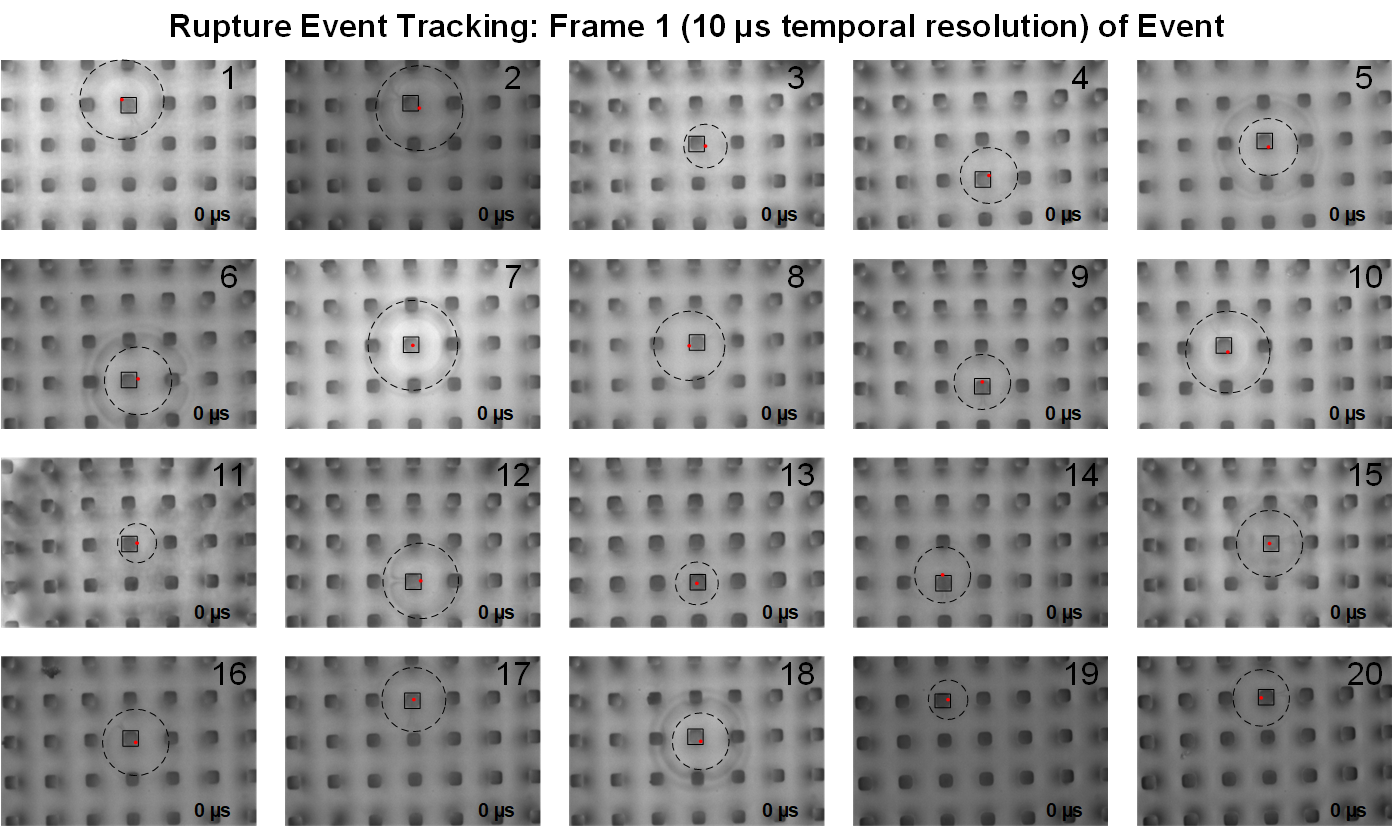


**Figure S5. Statistical Rupture Analysis of Bubble Approaching Micropillars (**$\boldsymbol{\alpha}$ **= 90%,** $\boldsymbol{w}$ **= 20 µm) over n = 20 Repeats.** The first frame, at 10 µs is used to capture the initial propagation wave, with the highlighted red-dot tracking the origin *via* a circle approximation.


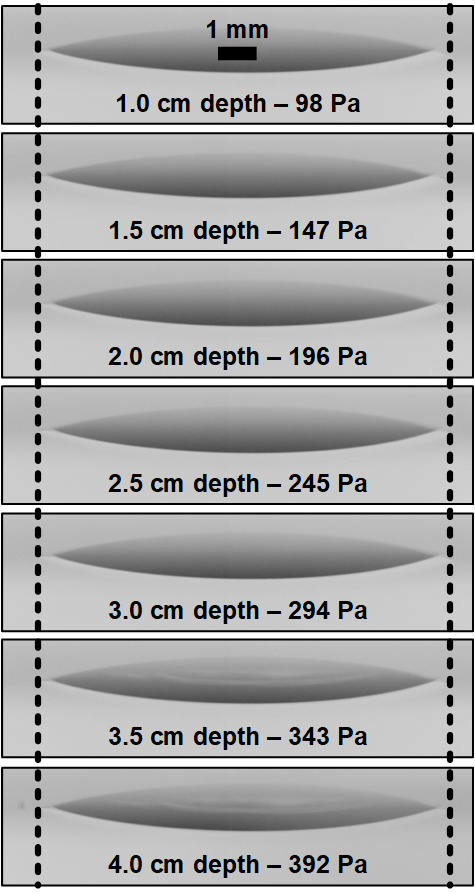


**Figure S6. Immersion Stability of a Hemi-Bubble on Micropillar Structures (**$\boldsymbol{w}$ **= 20 µm,** $\boldsymbol{s}$ **= 43 µm,** $\boldsymbol{h}$ **= 80 µm) Immersed at Various Depths with Increasing Pressure.** The bubble profile is likely unaffected because of the relative pressure imposed hydrostatically vs. the plastron pressure. Per the ideal gas law, $P_{1}V_{1}= P_{2}V_{2}$, the influence of even 400 Pa is too small to influence the volume ($V$). Compared to the plastron’s internal pressure of 101325 Pa, only *ca.* 0.4 % change in volume is expected. The dotted lines are drawn to provide a guide to the eye.

**
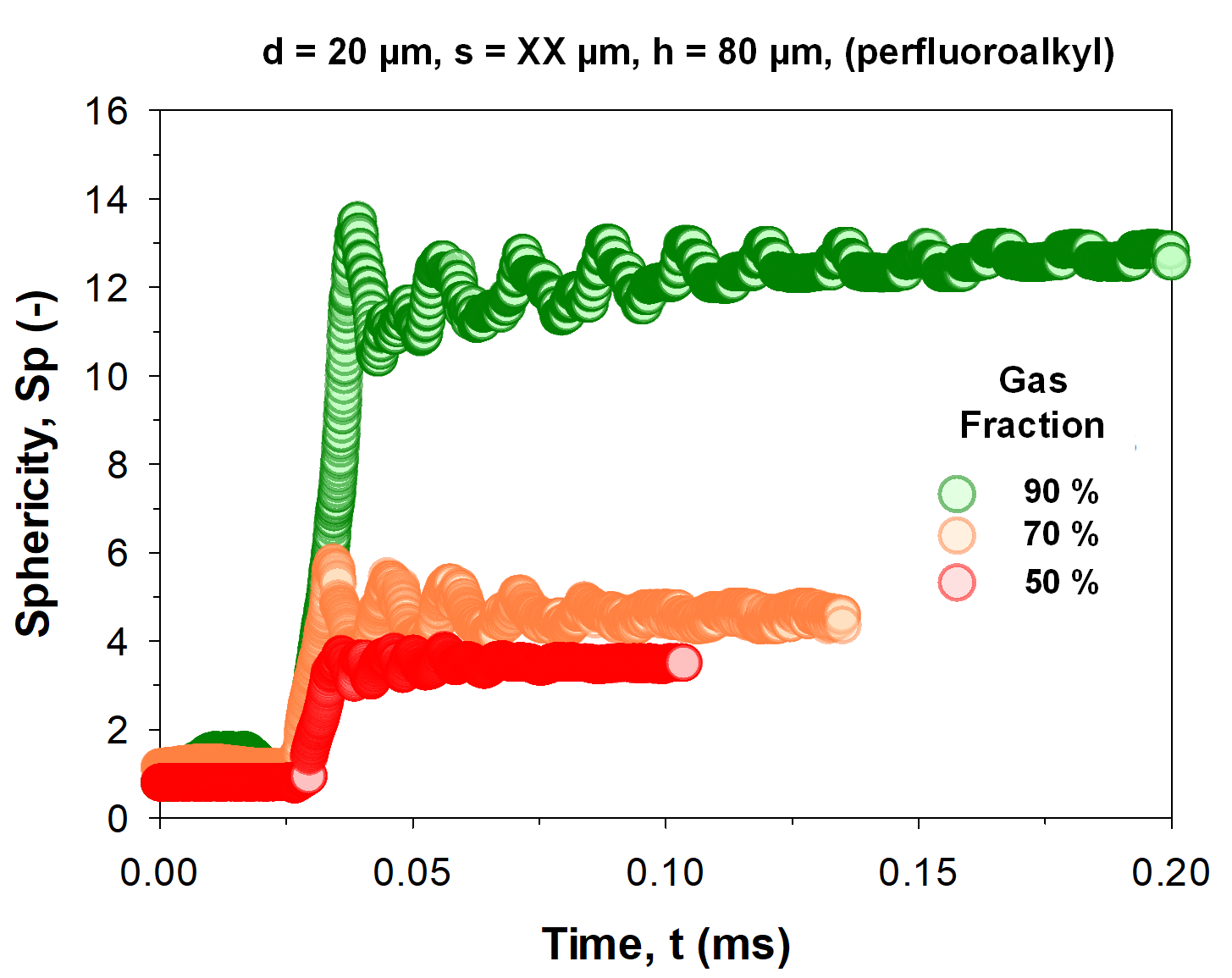
**

**Figure S7. Damping Effect of Hemi-Bubble Profile after Rupture, Expressed as Sphericity.** This is indicative of the experimentally observable contact line being anchored more strongly by the microstructured surfaces having a lower gas fraction (green to red).

**
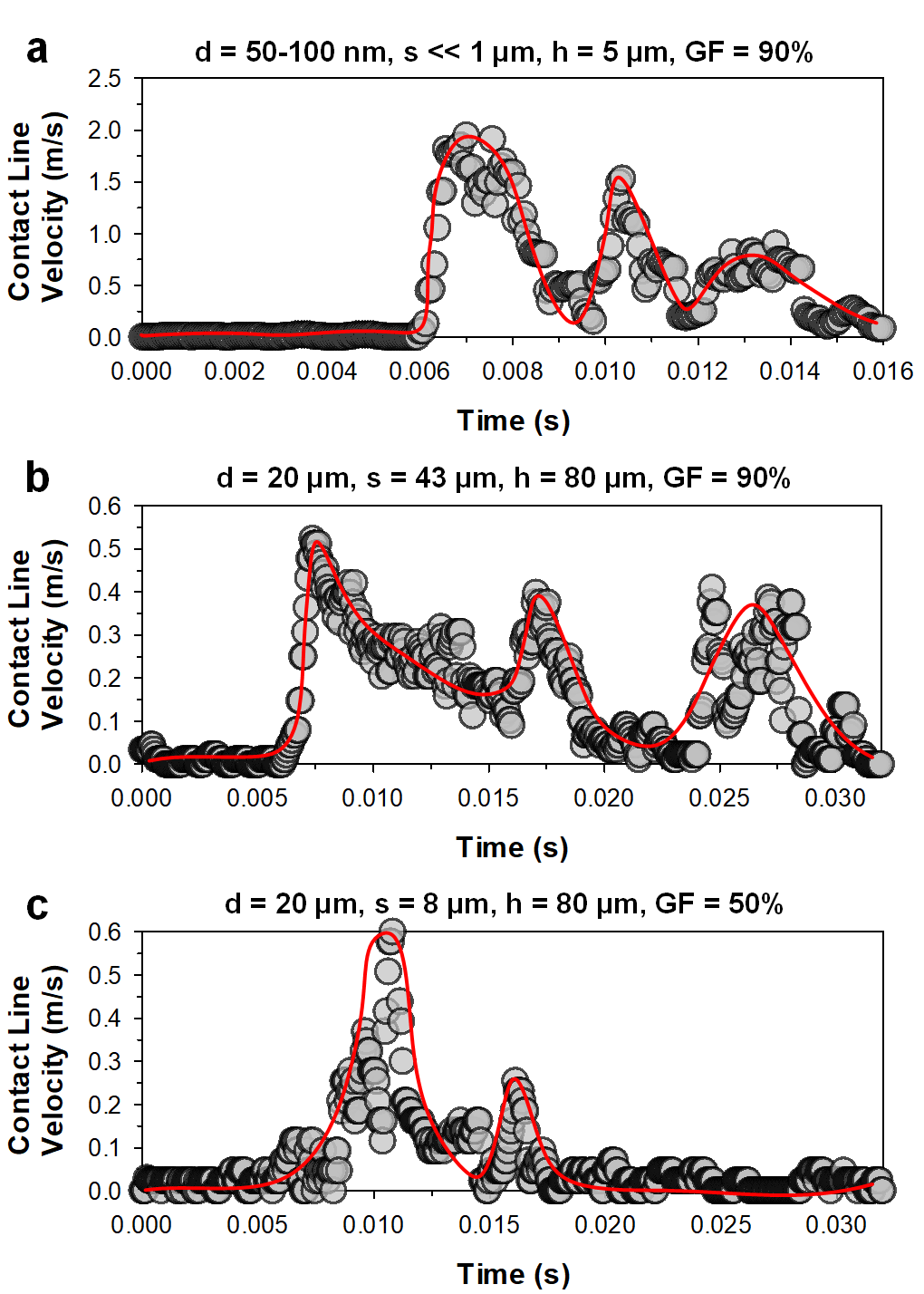
**

**Figure S8. Damping as Observed in Contact Line Motion (Velocity).** The contact line is tracked after rupture (at *ca.* 6 ms onwards). For hierarchical nanostructured surfaces, a) minimal damping is observed, as the rupture results in the first velocity spike of a spreading contact line, followed by a second spike induced by the jet-in of the ruptured bubble and subsequent fluctuations. For microstructured surfaces, a b) high gas fraction also allowed multiple velocity spikes, indicative of the repeated fluctuations in pushing the contact line further (less pinning). The c) lower gas fraction results in a rapid damping behavior after the initial spreading contact line.

**
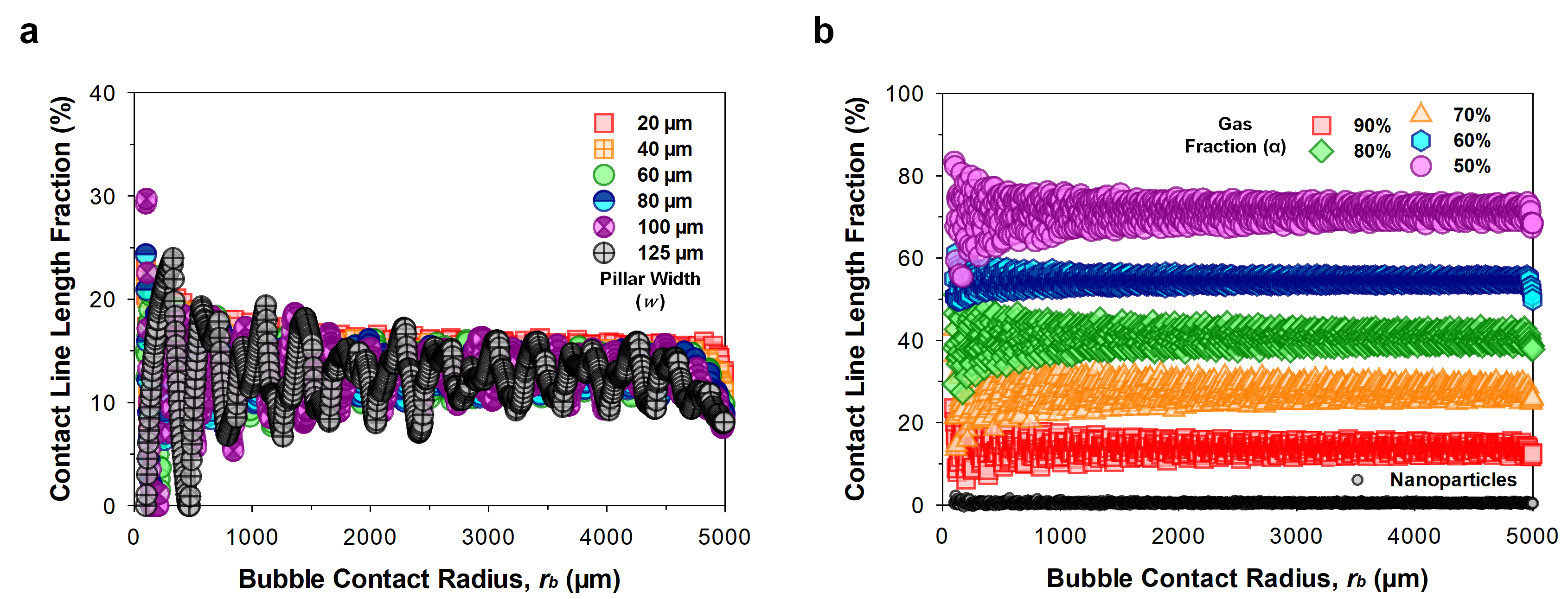
**

**Figure S9. Micro *vs.* Nanostructures: Simulating Contact Line Motion.** A simulated a,b) effective contact line length fraction (%) is mapped during the spread of the bubble (see manuscript, Figure 4). The effect behind a) feature size variation ($w$) and b) gas fraction (α) variation is presented. An b) experimentally mapped contact domain of a typical hierarchical nanostructured surface is also simulated for reference (small grey circles).


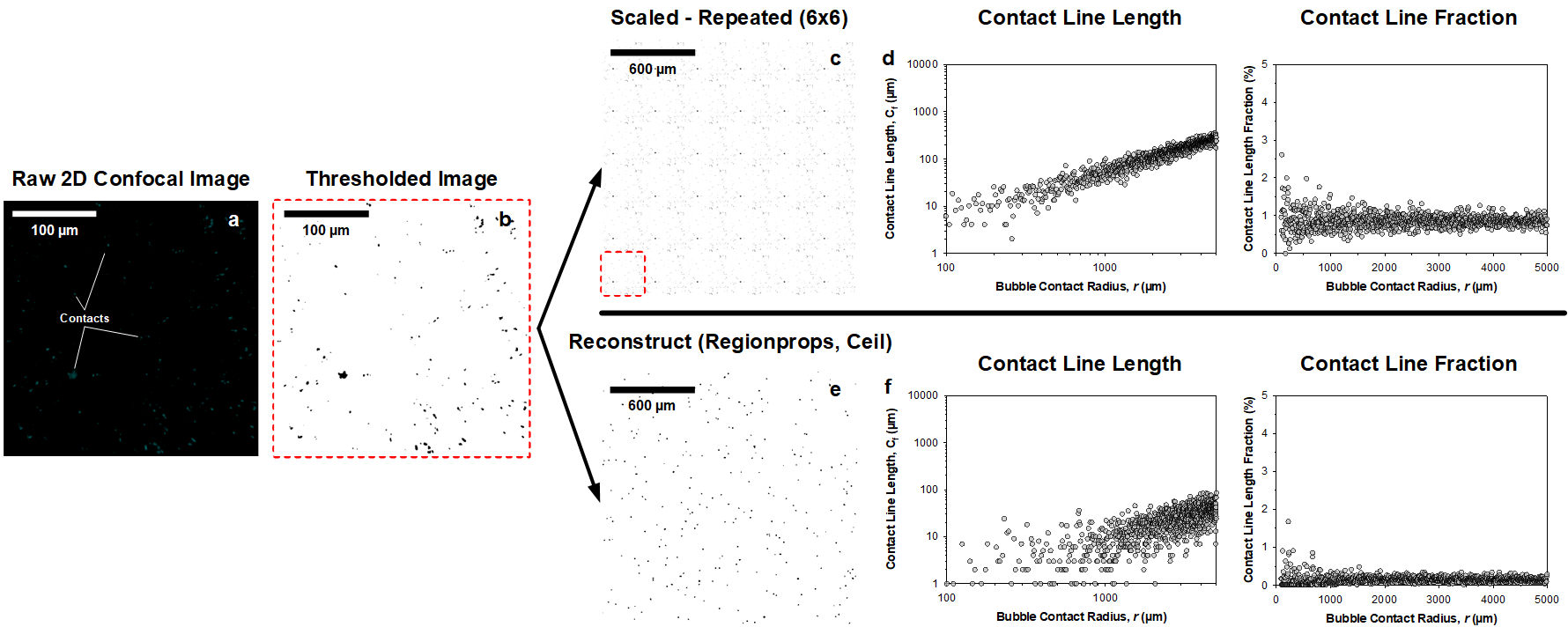


**Figure S10. Approximation of Liquid-Surface Contact by a) Confocal Imaging.^[^**[**^22^**](#_ENREF_22)**^]^** Microdroplet remnants from non-volatile organic liquids (ethylene glycol, dyed and represented in blue). The surfaces^[^[^22^](#_ENREF_22)^,^ [^23^](#_ENREF_23)^]^ for this reference^[^[^22^](#_ENREF_22)^]^ and this work are fabricated using the same synthesis conditions. The image (taken from raw data of *ACS Nano* **2020**, *4*, 3836) is first b) thresholded before processing *via* two methods: Firstly, c) the 300 µm x 300 µm is replicated/repeated and used to form a patchwork-like profile for over 12000 µm x 12000 µm needed for the bubble contact simulation. Secondly, the image can also be further processed using e) MATLAB (regionprops, “Size”) and used to reconstruct circular profiles from the original centroids. The distribution (spacing) and frequency (number) of locations is then processed into the final 10000 µm x 10000 µm needed for the bubble contact simulation. Based on the two methods employed, the c-d) patchwork-repeats appear to create more representative results *vs.* the e-f) use of further image processing tools (which appears to have removed some level of detail).

*Experimental (ACS Nano* **2020**, *4*, 3836*): “An inverted laser scanning confocal microscope (Leica TCS SP8) was used to observe remnant microdroplets. Ethylene glycol was dyed with ATTO 647-ester (λ_excitation_ = 620 nm, λ_emission_ = 647 nm) at a concentration of 10 μg/mL and rolled across the surfaces (in the order of 10 cm/s). A zoom factor of 1.0 and a line average of 2 (bidirectional) was used. A dry objective, Leica HC PL APO 10×, NA 0.4, was used. Lasers of 458 and 633 nm were used at ca. 15.4% power under the fluorescence mode. The XYZ stack was recorded over 200 steps as a 3D image. This process was completed within tens of seconds after the microdroplets were formed by a rolling drop.” Further details of the measurement can be found in ACS Nano****2020****, 4, 3836. This technique of contact estimation has been performed previously using optical microscopy and micropillars.^[^*[*^24^*](#_ENREF_24)*^,^* [*^25^*](#_ENREF_25)*^]^*


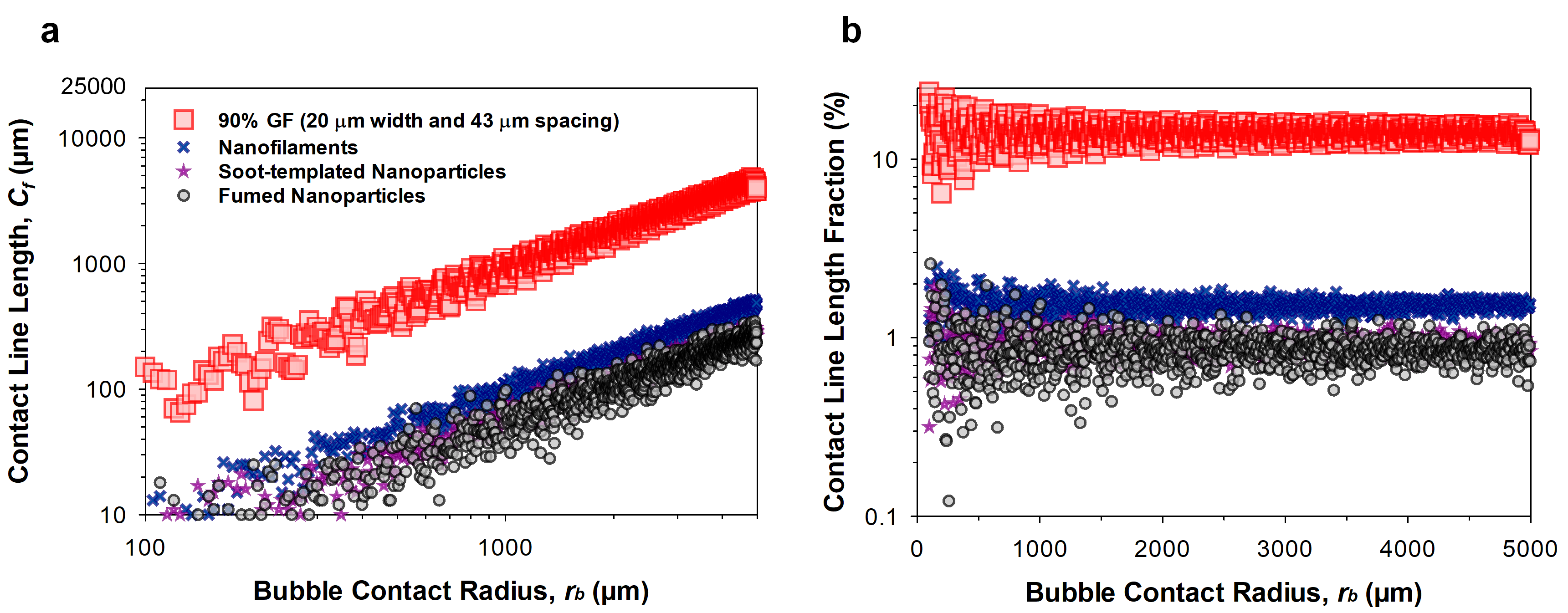


**Figure S11. Contact Profiling of Nanostructural Surfaces *via* Non-Volatile Ethylene Glycol Assisted Confocal Imaging (per Figure S10).** Actual replicated/repeated and scaled profiles were used. The overall a) contact line length and b) contact line fraction is illustrated for nanofilaments (blue crosses), soot-templated nanoparticles (purple stars) and spray-deposited fumed nanoparticles (grey circles). The last variant is that which was used in this study. The optimal microstructured surfaces is included (90% GF, $w$ = 20 µm, $s$ = 43 µm) for reference (red squares).


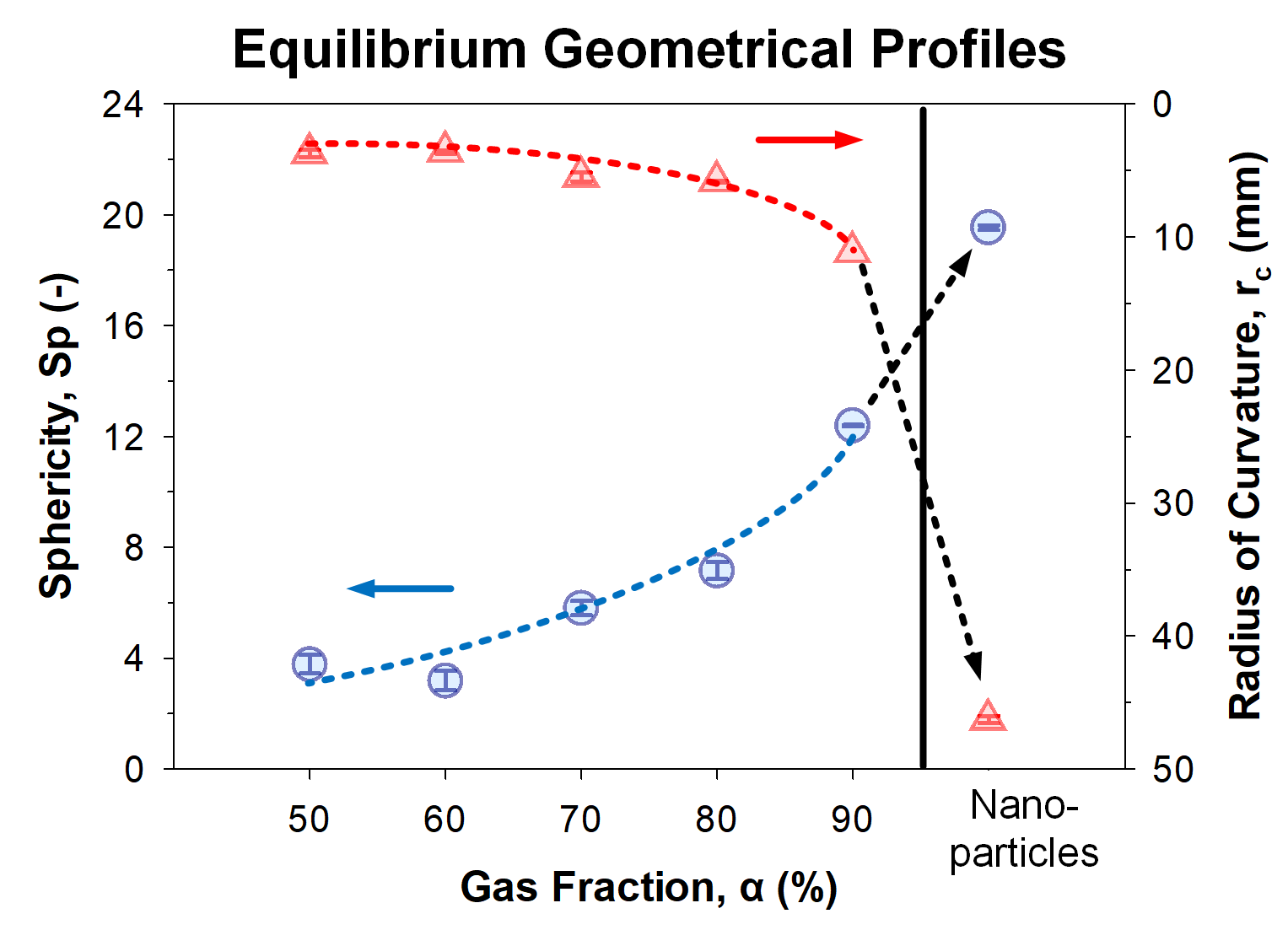


**Figure S12. Equilibrium Profiles Post-Rupture: Influence of Gas Fraction (**$\boldsymbol{\alpha}$**).** With variable gas fraction, the equilibrium bubble sphericity, $Sp$ (1 = perfect sphere) and radius of curvature, $r_{c}$ appears to both increase with increasing $\alpha$ (up to 90%), with nanostructures sharply increasing this trend.


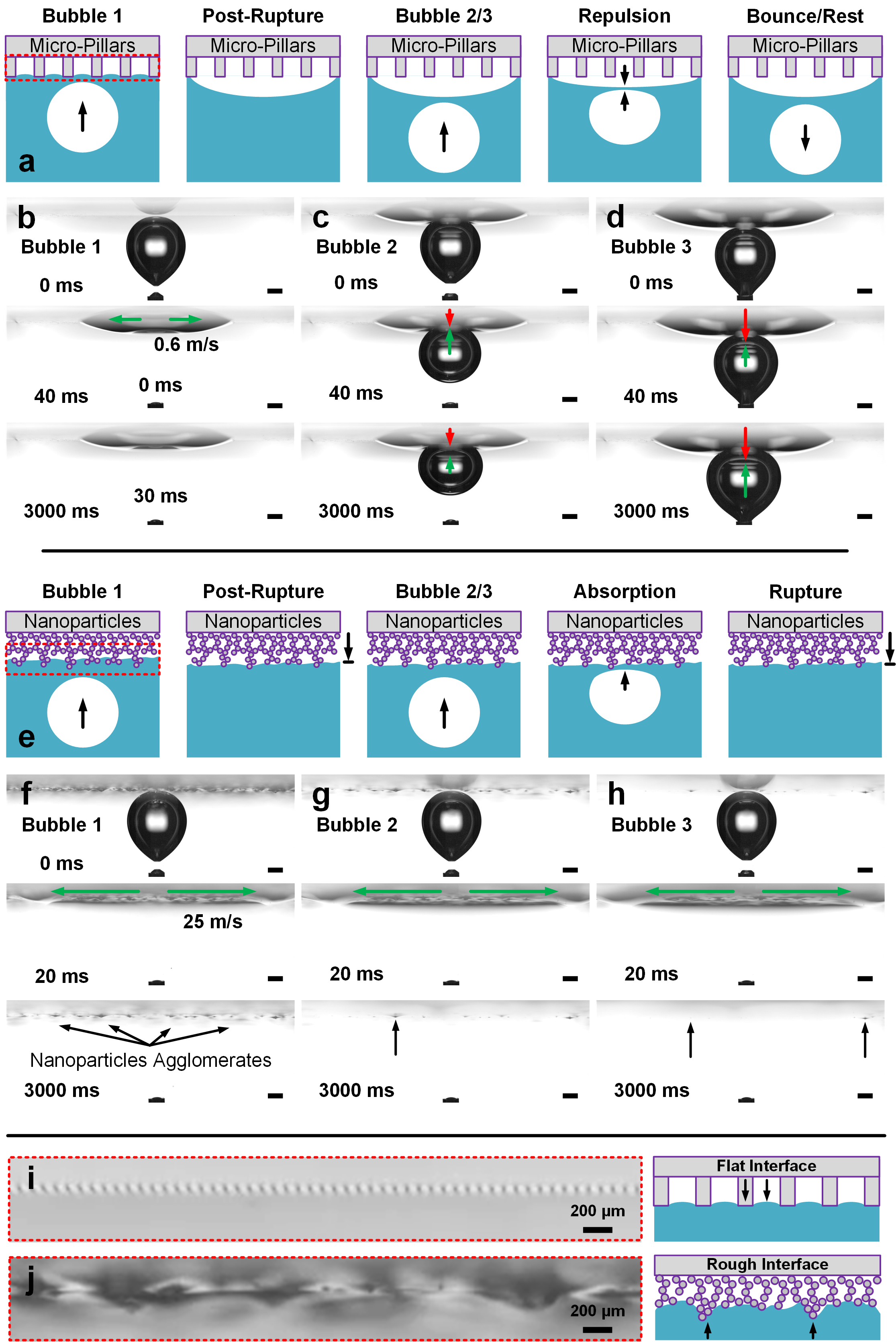


**Figure S13. Micro *vs.* Nanostructures: Multi-Bubble Rupture-and-Absorption.** a) Microstructured surfaces can execute fast bubble rupture. However, the inability to spread and absorb air impedes secondary bubble capture. b) After bubble rupture (*i.e.* bubble 1), the contact line is halted at a finite distance, forming a hemi-bubble that prevents plastron-induced rupture of c-d) secondary incoming bubbles, forcing them to rupture at timescales of nearly 2 orders of magnitude slower (3-5 s *vs.* 20 ms) *via* bubble-to-bubble rupture. e) Hierarchical nanostructured surfaces can rupture-and-absorb multiple bubbles without any loss in performance. f-h) Bubble rupture is kept rapid (< 10 ms) regardless of secondary incoming bubbles due to a loosely pinned contact line. Magnified photographic images and schematics of the i) microstructured plastron and the j) hierarchical nanostructured plastron.


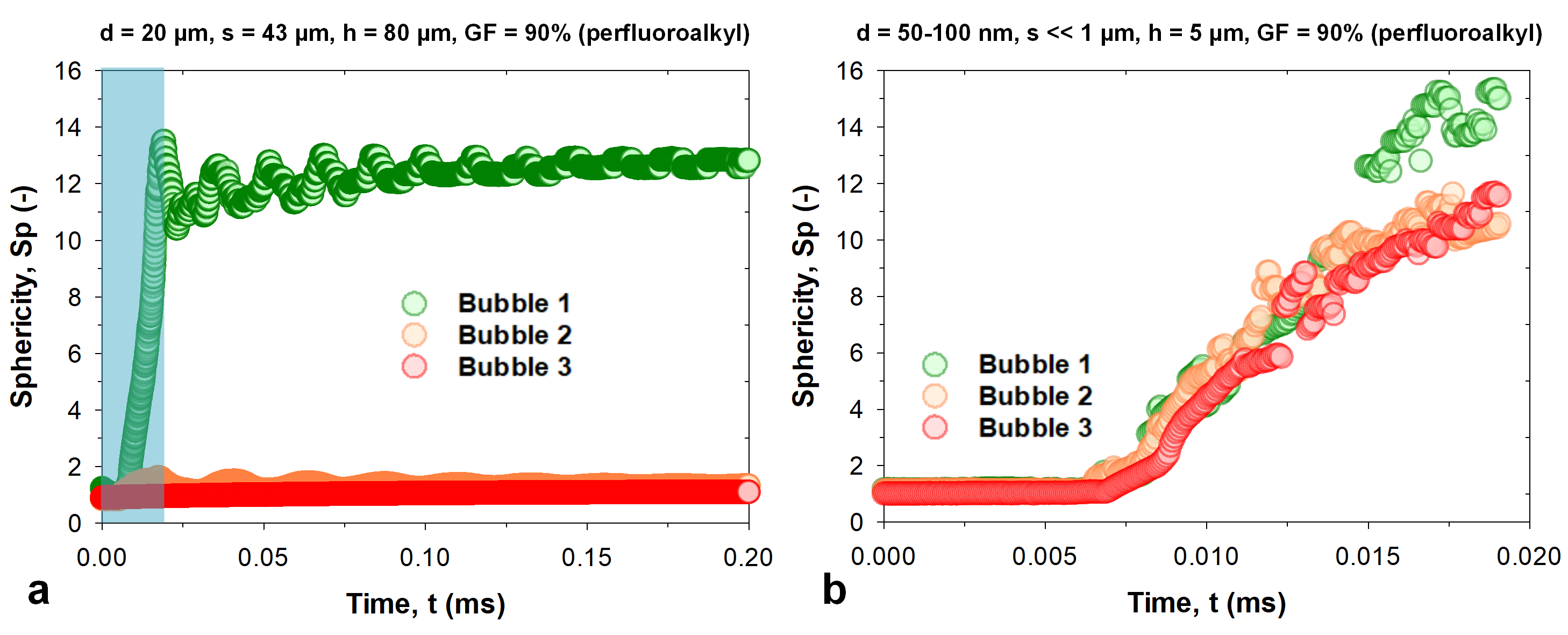


**Figure S14. Sphericity of Multiple Contacting Bubbles with a) Microstructured and b) Hierarchical Nanostructured Surfaces.** A sphericity of 1 denotes a circular bubble (whole, unruptured) while a high sphericity indicates rupture (*i.e.* no longer a sphere). Green: Bubble 1, Orange: Bubble 2, and Red: Bubble 3. The blue domain highlighted in a) represents the entire timescale of b).

**
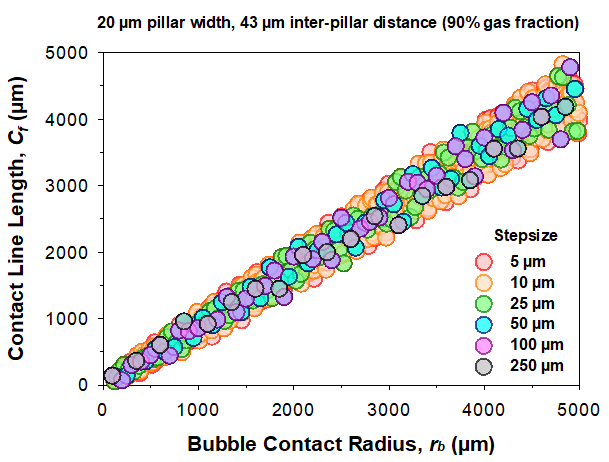
**

**Figure S15. Contact Line Modelling (Insensitive to Step Size).** To provide the greatest detail, a 5 µm step-size is chosen for representation.


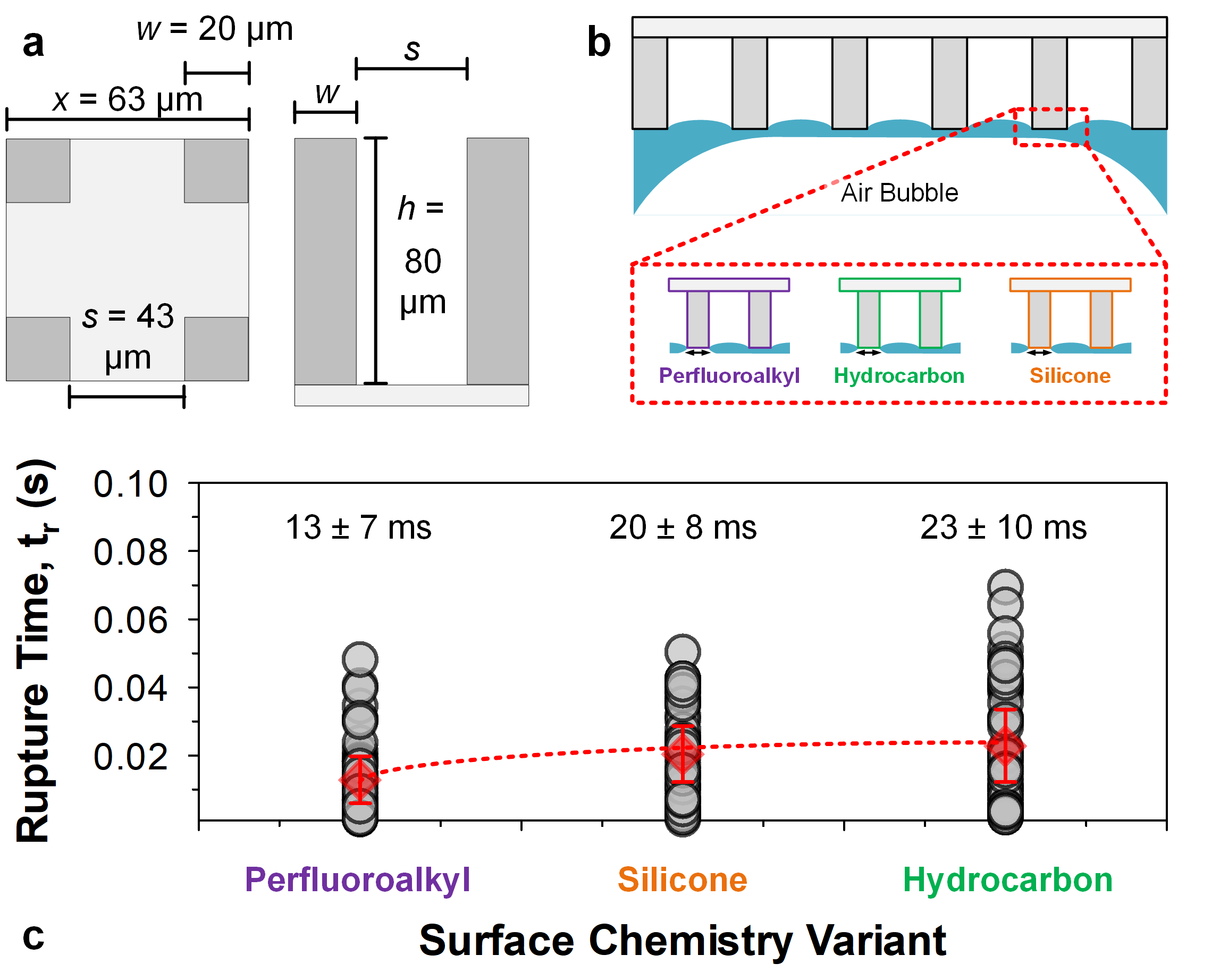


**Figure S16. Mechanism: Surface Chemistry Variation. Optimal** a) Optimally developed micropillars (20 µm width, 43 µm wall-to-wall separation, 90% gas fraction) were tested across b) three surface functionalization regimes: 1) perfluoroalkyl, 2) silicone, and 3) hydrocarbon. Rupture timing assessment of these different surface chemistries in milliQ water, 18.2 MΩ.cm show c) a weak correlation, with error range of each type overlapping.

**References**

[1] B. Liu, R. Manica, Q. Liu, E. Klaseboer, Z. Xu, G. Xie, *Phys. Rev. Lett.* **2019**, *122*, 194501.

[2] W. S. Y. Wong, A. Naga, L. Hauer, P. Baumli, H. Bauer, K. I. Hegner, M. D’Acunzi, A. Kaltbeitzel, H.-J. Butt, D. Vollmer, *Nat. Commun.* **2021**, *12*, 5358.

[3] M. S. Shah, C. R. Kleijn, M. T. Kreutzer, V. van Steijn, *Phys. Rev. Fluids* **2021**, *6*, 013603.

[4] E. Chatzigiannakis, N. Jaensson, J. Vermant, *Curr. Opin. Colloid Interface Sci.* **2021**, *53*, 101441.

[5] D. Y. C. Chan, E. Klaseboer, R. Manica, *Soft Matter* **2011**, *7*, 2235-2264.

[6] M. Kappl, H. J. Butt, *Surface and interfacial forces*, Wiley, **2018**.

[7] D. Tabor, R. H. S. Winterton, *Proc. Math. Phys. Eng. Sci.* **1969**, *312*, 435-450.

[8] L. Rapoport, T. Emmerich, K. K. Varanasi, *Adv. Mater. Interfaces* **2020**, *7*, 1901599.

[9] Y. Xing, X. Gui, L. Pan, B.-E. Pinchasik, Y. Cao, J. Liu, M. Kappl, H.-J. Butt, *Adv. Colloid Interface Sci.* **2017**, *246*, 105-132.

[10] K. I. Hegner, W. S. Y. Wong, D. Vollmer, *Adv. Mater.* **2021**, *33*, 2101855.

[11] Y. J. Kim, A. Lim, J. M. Kim, D. Lim, K. H. Chae, E. N. Cho, H. J. Han, K. U. Jeon, M. Kim, G. H. Lee, G. R. Lee, H. S. Ahn, H. S. Park, H. Kim, J. Y. Kim, Y. S. Jung, *Nat. Commun.* **2020**, *11*, 4921.

[12] G. Liu, W. S. Y. Wong, M. Kraft, J. W. Ager, D. Vollmer, R. Xu, *Chem. Soc. Rev.* **2021**, 10.1039/d1cs00258a.

[13] D. Bonn, J. Eggers, J. Indekeu, J. Meunier, E. Rolley, *Rev. Mod. Phys.* **2009**, *81*, 739-805.

[14] P. G. de Gennes, *Rev. Mod. Phys.* **1985**, *57*, 827-863.

[15] J. H. Snoeijer, B. Andreotti, *Annu. Rev. Fluid Mech.* **2013**, *45*, 269-292.

[16] A. Carlson, M. Do-Quang, G. Amberg, *J. Fluid Mech.* **2011**, *682*, 213-240.

[17] H.-J. Butt, N. Gao, P. Papadopoulos, W. Steffen, M. Kappl, R. Berger, *Langmuir* **2017**, *33*, 107-116.

[18] D. F. James, *J. Fluid Mech.* **1974**, *63*, 657-664.

[19] B. V. Derjaguin, *Dok. Akad. Nauk SSSR*, *51*, 517-520.

[20] W. S. Y. Wong, M. S. Kiseleva, S. Zhou, M. Junaid, L. Pitkänen, R. H. A. Ras, *Adv. Mater.* **2023**, *n/a*, 2300306.

[21] W. S. Y. Wong, P. Bista, X. Li, L. Veith, A. Sharifi-Aghili, S. A. L. Weber, H.-J. Butt, *Langmuir* **2022**, *38*, 6224-6230.

[22] W. S. Y. Wong, T. P. Corrales, A. Naga, P. Baumli, A. Kaltbeitzel, M. Kappl, P. Papadopoulos, D. Vollmer, H.-J. Butt, *ACS Nano* **2020**, *14*, 3836-3846.

[23] W. S. Y. Wong, *Nano Lett.* **2019**, *19*, 1892-1901.

[24] H.-J. Butt, I. V. Roisman, M. Brinkmann, P. Papadopoulos, D. Vollmer, C. Semprebon, *Curr. Opin. Colloid Interface Sci.* **2014**, *19*, 343-354.

[25] B. Su, S. Wang, J. Ma, Y. Song, L. Jiang, *Adv. Funct. Mater.* **2011**, *21*, 3297-3307.
